# Nanostars Carrying Multifunctional Neurotrophic Dendrimers Protect Neurons in Preclinical In Vitro Models of Neurodegenerative Disorders

**DOI:** 10.1021/acsami.2c14220

**Published:** 2022-10-11

**Authors:** Corinne Morfill, Stanislava Pankratova, Pedro Machado, Nathalie K. Fernando, Anna Regoutz, Federica Talamona, Alessandra Pinna, Michal Klosowski, Robert J. Wilkinson, Roland A. Fleck, Fang Xie, Alexandra E. Porter, Darya Kiryushko

**Affiliations:** † Department of Materials and London Centre for Nanotechnology, Imperial College, Exhibition Road, LondonSW7 2AZ, UK; ‡ Department of Neuroscience, Faculty of Health and Medical Sciences, University of Copenhagen, Copenhagen2200N, Denmark; § Comparative Paediatrics and Nutrition, Department of Veterinary and Animal Sciences, Faculty of Health and Medical Sciences, University of Copenhagen, Copenhagen2200N, Denmark; ∥ Centre for Ultrastructural Imaging, Kings College London, LondonSE1 1UL, UK; ⊥ Department of Chemistry, University College London, 20 Gordon Street, LondonWC1H 0AJ, UK; # The Francis Crick Institute, LondonNW11 AT, UK; ∇ Imperial College, Exhibition Road, LondonSW7 2AZ, UK; ○ Centre for Neuroinflammation and Neurodegeneration, Imperial College London, Hammersmith Hospital Campus, Burlington Danes Building, 160 Du Cane Road, LondonW12 0NN, UK; ◆ Experimental Solid State Physics Group, Department of Physics, Imperial College, Exhibition Road, LondonSW72AZ, UK

**Keywords:** S100A4, peptides, mimetic, neuron, gold nanostar, neuroprotection

## Abstract

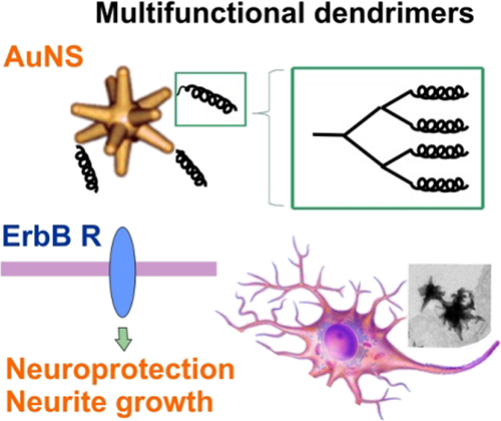

A challenge in neurology is the lack of efficient brain-penetrable neuroprotectants targeting multiple disease mechanisms. Plasmonic gold nanostars are promising candidates to deliver standard-of-care drugs inside the brain but have not been trialed as carriers for neuroprotectants. Here, we conjugated custom-made peptide dendrimers (termed H3/H6), encompassing motifs of the neurotrophic S100A4-protein, onto star-shaped and spherical gold nanostructures (H3/H6-AuNS/AuNP) and evaluated their potential as neuroprotectants and interaction with neurons. The H3/H6 nanostructures crossed a model blood–brain barrier, bound to plasma membranes, and induced neuritogenesis with the AuNS, showing higher potency/efficacy than the AuNP. The H3-AuNS/NP protected neurons against oxidative stress, the H3-AuNS being more potent, and against Parkinson’s or Alzheimer’s disease (PD/AD)-related cytotoxicity. Unconjugated S100A4 motifs also decreased amyloid beta-induced neurodegeneration, introducing S100A4 as a player in AD. Using custom-made dendrimers coupled to star-shaped nanoparticles is a promising route to activate multiple neuroprotective pathways and increase drug potency to treat neurodegenerative disorders.

## Introduction

At present, therapeutic opportunities for neurological disorders (NDs) are severely limited and are mostly directed toward symptom relief. The major challenge is the complexity of ND pathophysiology involving several contributing factors such as disruption of neuronal membranes, inflammation, oxidative stress, and excitotoxicity. Current therapies predominantly address isolated ND aspects, alleviating clinical symptoms but failing to halt disease progression. There is an urgent clinical need to devise strategies to reduce neuronal damage by targeting multiple ND mechanisms. However, there is a lack of easily manufactured multifunctional compounds that could cross the blood–brain barrier (BBB) and protect neurons with minimal side effects. Nanoneurotherapeutics are a promising avenue as they can be engineered small enough to cross the BBB yet with high surface area to perform multiple orthogonal functions including delivery of high drug loads, cell targeting, and bioimaging.^1−3^

**Figure 1:**
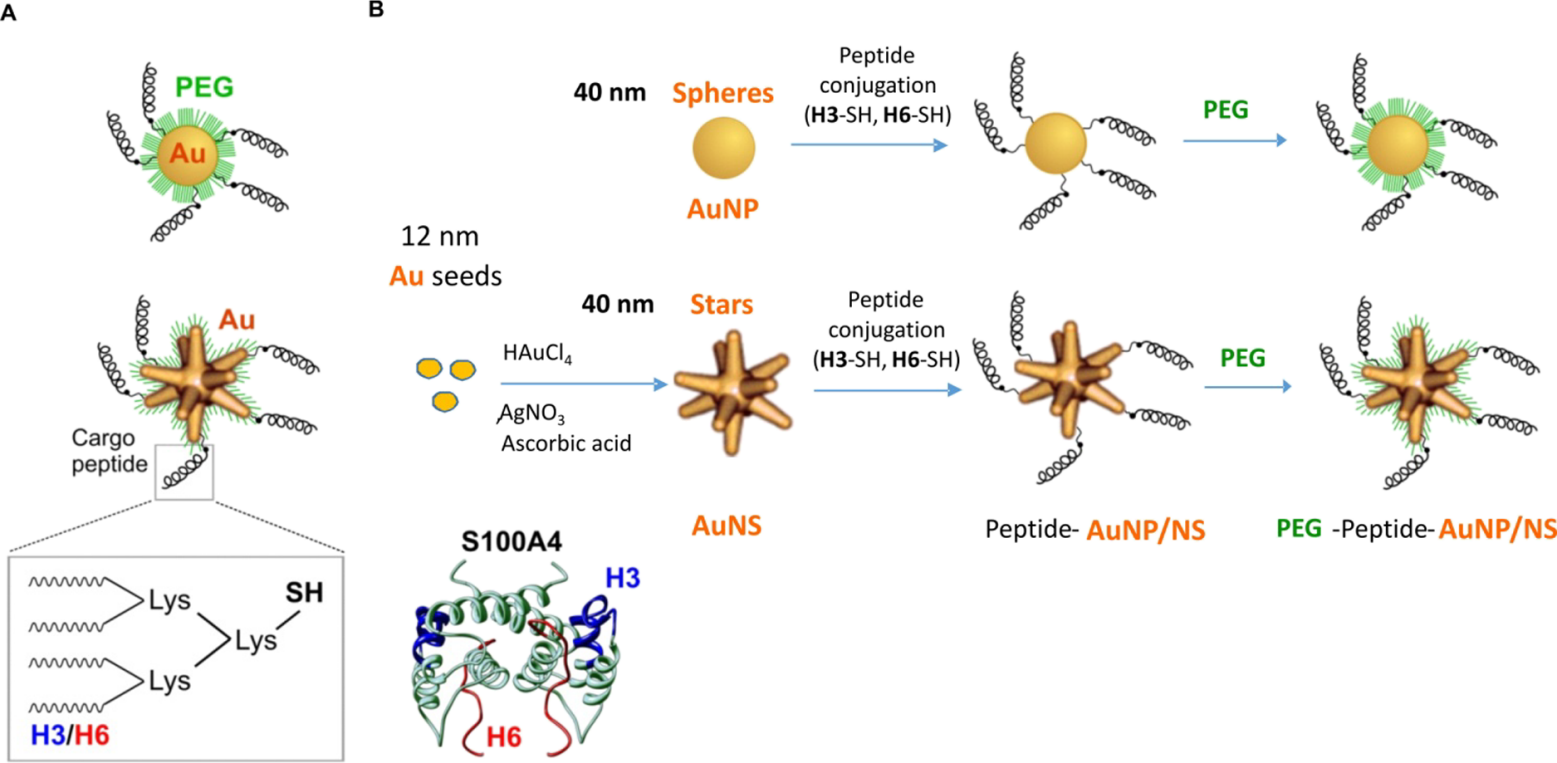
Synthesis of (+/–PEG)-H3/H6-conjugated gold nanostructures. (A) Schematic representation of PEG- and peptide-conjugated spherical and star-shaped Au nanostructures (AuNP/AuNS). Inset, cargo peptides (H3/H6) synthesized as tetramers composed of four monomers coupled to a lysine backbone. Thiol modification for conjugation to gold (-SH) resides on a lysine backbone not affecting neuroactive motifs. The location of the H3 and H6 motifs in the 3D structure of the parent protein (S100A4) is shown. (B) Scheme summarizing the fabrication protocol and structures synthesized at each step of the protocol to fabricate (+/–PEG)-H3/H6-AuNP/AuNS.

We have previously designed two custom peptide dendrimers (H3/H6, Figure 1) encompassing active motifs of the neurotrophic protein S100A4 that we have identified.^4,5^ Similar to the parent protein, the peptides promoted neuritogenesis, reduced oxidative stress and excitotoxicity via multiple pathways, and protected neurons in animal models of brain trauma, epilepsy, and peripheral neuropathy.^4−6^ The peptides are good candidates for multipurpose neuroprotectants because of their ability to target multiple neuroprotective mechanisms. Importantly, their dendrimeric structure makes these peptides highly efficient and easy to modify without affecting the bioactive motifs. However, a limitation is that despite being detected in cerebrospinal fluid after systemic administration, they display limited brain penetration and circulation half-life.^4^ We have shown that gold nanostars can cross the BBB at high (up to 40%) rates and closely interact with the plasma membrane of cells, making nanostars a promising drug delivery vehicle to treat neurodegenerative disorders.^7^ We have also engineered spherical AuNPs that selectively antagonize extrasynaptic NMDA receptors without affecting synaptic function and protect neurons from excitotoxicity.^8^ Here, we conjugated H3/H6 onto star-shaped and spherical gold nanostructures (H3/H6-AuNS/AuNP) to design novel neuroprotectants and investigate whether H3/H6-AuNSs are more effective as neurotrophic agents than their spherical counterparts. This mechanistic insight could be used to inform design optimizations for future testing in vivo.

A multifunctional trophic factor in the nervous system, the S100A4 protein,^4^ is markedly (over)expressed in rodent and human brains after injury and during neuropathology.^4,9−11^ S100A4 signals through the interleukin-10 and ErbB receptors,^4,6^ which are involved in neuronal plasticity in brain disorders promoting neurogenesis, neuronal differentiation, and survival^12−16^ and mediating neuroprotection in models of cerebral ischemia and Alzheimer’s and Parkinson’s diseases (AD/PD).^13,17−21^ Accordingly, we have shown that S100A4 induces neurite extension^22,23^ and protects neurons against oxidative stress, apoptosis, and excitotoxicity in vitro and in vivo, thus representing a broad spectrum CNS protectant.^4^ The beneficial effects of S100A4 are reproduced by its two peptide mimetics, H3 and H6, encompassing the neuroactive motifs of the protein. The H3 peptide is highly homologous within the S100 family, while the H6 peptide (C-terminal of S100A4) shares little homology with other S100 members (’unique’ motif)^4,5^ (Figure 1). The peptides bind to ErbB, induce neurite outgrowth from hippocampal, dopaminergic, and motor neurons,^4−6^ and protect neurons in cell and animal models of brain trauma and excitotoxicity^4^ as well as against PD-associated neurotoxicity in vitro^6^ and genetically induced peripheral neuropathy in vivo*.*^5^ In contrast to the parent protein, the peptides do not affect cell proliferation or motility^6^ and thus have a specific neurotrophic function.

Little is known about the role of ErbBs in PD and AD; however, ErbB activation improves neuronal survival and synaptic function in AD models,^13,17,24^ and ErbB4 is highly phosphorylated in the neurons of the AD human brain and of APP/PS1 mice, a model of early onset AD.^25^ Furthermore, oligomeric amyloid beta (Aβ)—a major inducer of neurotoxicity in AD—binds to ErbB4,^26^ presumably increasing Aβ insertion into neuronal membranes. In our two recent studies, Aβ disrupted the membrane structure in cell lines and hippocampal neurons, and this deleterious effect was counteracted by the H3-peptide,^27,28^ representing an important, though overlooked, aspect of neuroprotection. Thus, the S100-derived peptides target multiple neuroprotective mechanisms by both launching intracellular signaling cascades and, for AD, counteracting the effects of Aβ on membrane structures. Therefore, these peptides may be effective for a range of neurological disorders.

Small peptides offer several advantages over protein/growth factor-based therapeutics since they (i) specifically encompass neuroactive motifs of the parent protein, thus minimizing potential side effects; (ii) are low cost and readily producible; and (iii) can be easily chemically modified.

Our custom nanoplatform comprises multifunctional S100-derived peptide dendrimers conjugated to gold nanospheres/stars and optionally coated with polyethyleneglycol (PEG) to improve compound stability and half-life.^29^ We evaluate these nanostructures in cell models of neurological disorders and of a blood–brain barrier (BBB), assess their biocompatibility in vivo, and investigate the effects of the nanostructure geometry on biological responses. We used transmission electron microscopy and electron tomography to compare the interaction of the star and spherical nanostructures with cell membranes to understand the mechanisms by which the spikes of the nanostars increase their cell binding properties.

## Results

### Synthesis and Physicochemical Characterization of H3/H6-AuNP/AuNS

**Figure 2:**
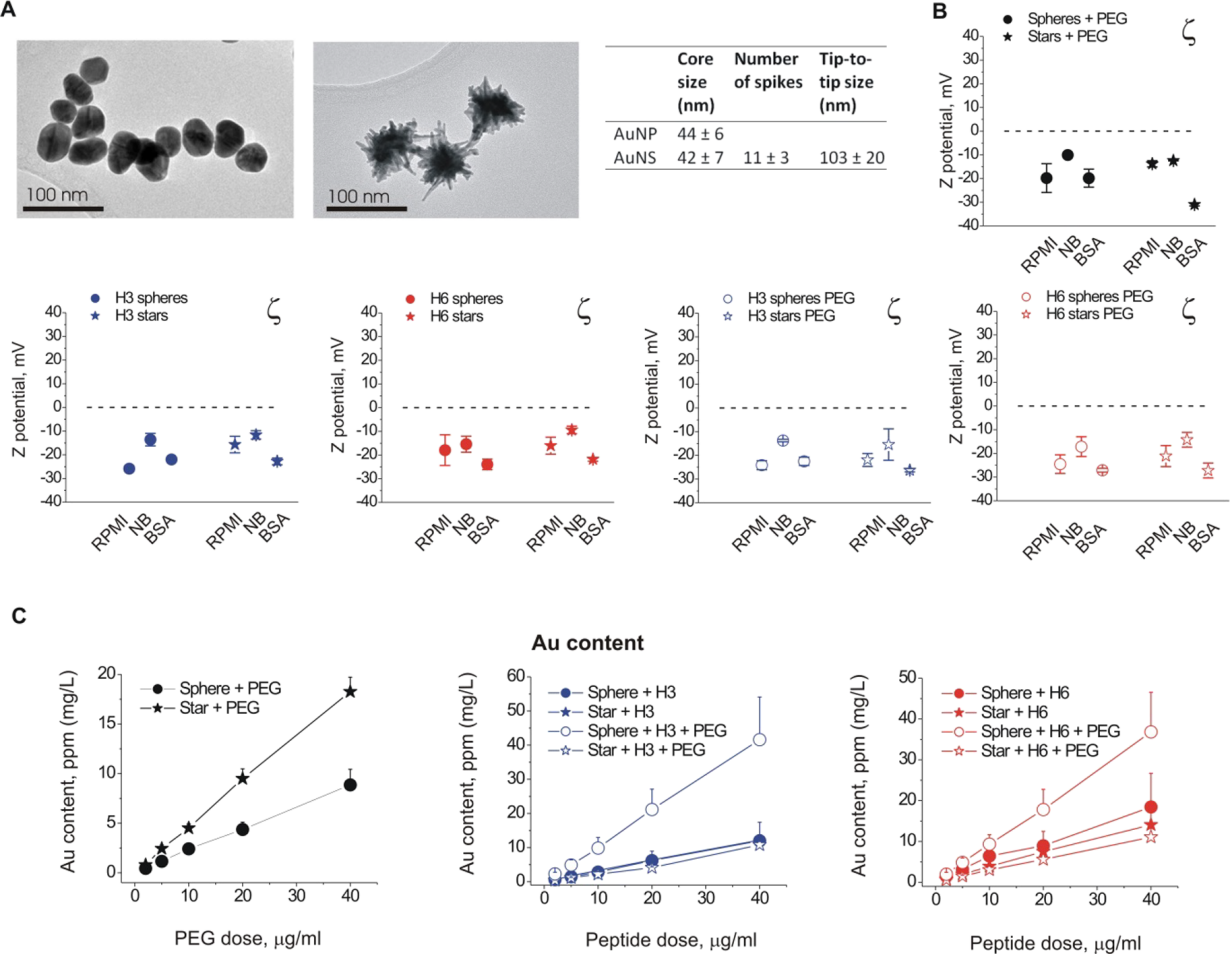
Characterization of the (+/–PEG)-H3/H6-conjugated gold nanostructures. (A) Low-magnification TEM image of the AuNP/NS structures (left) and morphological characteristics of gold nanostructures (AuNP/NS), determined by TEM (right, *n* = 40–60, means ± SD, fitted to lognormal distribution). (B) Zeta potential of the Au NP/NS in various media (RPMI, RPMI 1640 growth medium; NB, Neurobasal neuronal growth medium; BSA, 1% bovine serum albumin); (C) quantification of gold content per PEG or peptide dose in the (+/–PEG)-H3/H6-AuNP/AuNS. (B,C) three independent experiments; values are shown as means ± SEM.

The fabrication protocol for the H3/H6-nanostructures is schematically shown in Figure 1 (see Materials and Methods for details). H3/H6-AuNSs were synthesized *via* a surfactant-free seed-mediated wet chemical approach using 12 nm Au seeds as previously described.^2,30^ A surfactant-free method does not use polymers or surfactants in synthesis and thus results in highly biocompatible AuNS that can be easily functionalized with cargo drugs for biomedical applications. Both spherical (AuNP) and star-shaped (AuNS) nanostructures were prepared. AuNSs had the conventional multibranched morphology of a spherical core with multiple protruding spikes (TEM images, Figure 2A). The morphological properties of the AuNP/NS including core size, the number of visible spikes, and the overall (tip-to-tip) AuNS size were quantified from multiple transmission electron microscopy (TEM) images (Figure 2A). The ellipticity index of AuNP was 1.21, which is within the range for spherical nanoparticles (1.05–1.25)^31^ and consistent with a previous report showing that the AuNP had an ellipticity of 1.16 for the citrate reduction method^32^ (see Materials and Methods for details). The sphericity indices calculated as described in Materials and Methods were 0.97 ± 0.03 and 0.22 ± 0.11 for AuNP and AuNS (mean ± SD), respectively.

The AuNP/NS surface was coated with either thiol-modified H3/H6 alone or with a mixture of thiol-modified peptides and alkyl polyethylene glycol (PEG2000) thiols (see Materials and Methods for details). In all peptide formulations, thiol modifications were localized in the lysine backbone of each peptide, thus not affecting neuroactive peptide sequences (Figure 1A, bottom). PEGylation of polypeptide drugs is widely used in pharmaceutics, protecting them from degradation by proteolytic enzymes, preventing generation of neutralizing antibodies, and increasing compound half-life.^33^ In agreement with previous reports,^34^ naked AuNPs had a UV–vis absorption maximum around 530 nm, and functionalization with thiol-modified peptides resulted in a minor (ca. 3 nm) shift of the absorption maximum (Figure S1).^34^ No major spectral changes were observed in H3/H6-AuNP/NS, indicating that the nanostructures did not aggregate following peptide functionalization. UV–vis spectrophotometry of the nanostructure’s stability in deionized water over 3 weeks (Figure S2b, H3/H6 nanospheres) showed that less than 2% of the AuNP-immobilized peptides was released after 18 days, confirming the stability of our nanocompounds, and the H3 nanospheres (ca. 0.5% peptide released) appeared more stable than the H6 nanospheres (ca. 1.4% peptide released). Accordingly, the dynamic light scattering (DLS) analysis of H3 nanospheres (Figure S2c) showed a slight increase of hydrodynamic diameter over 16 days (from 76.4 ± 0.4 nm at day 0 to 101.6 ± 1.7 nm at day 16) but no detectable peptide dissociation from the nanospheres.

The zeta potential of the coated AuNP/NS at pH 7 was mildly negative in the RPMI 1640 and neuronal Neurobasal media as well as in the 1% bovine serum albumin solution (BSA, Figure 2B). The zeta potentials did not vary significantly depending on the media types, particle geometry, or PEGylation, remaining in the range of −30 to −10 mV.

To estimate the relative peptide load of our particles, we measured the gold content in the samples of peptide and/or PEG-conjugated AuNP/NS using inductively coupled plasma–optical emission spectrometry (ICP-OES). Since no detectable amounts of free peptide/PEG were present in any of the AuNP/NS suspensions, the ICP measurements provided a good estimate of the amount of gold corresponding to a given peptide dose for AuNP/NS and thus of the efficiency of functionalization. The unconjugated spherical AuNP bound PEG more efficiently than the AuNS (lower Au content for a given PEG dose, Figure 2C, left). There was no significant difference between Au content for peptide-conjugated AuNP formulations of different geometries with the exception of spherical H3/H6-PEG-AuNP, which had a lower functionalization efficiency (Figure 2C, middle and right). Thus, PEGylated nanostars had more peptide immobilized on their surface than their spherical counterparts of the same mass.

### Evaluation of the H3/H6-PEG-AuNP/NS Formulations by X-ray Photoelectron Spectroscopy

**Figure 3:**
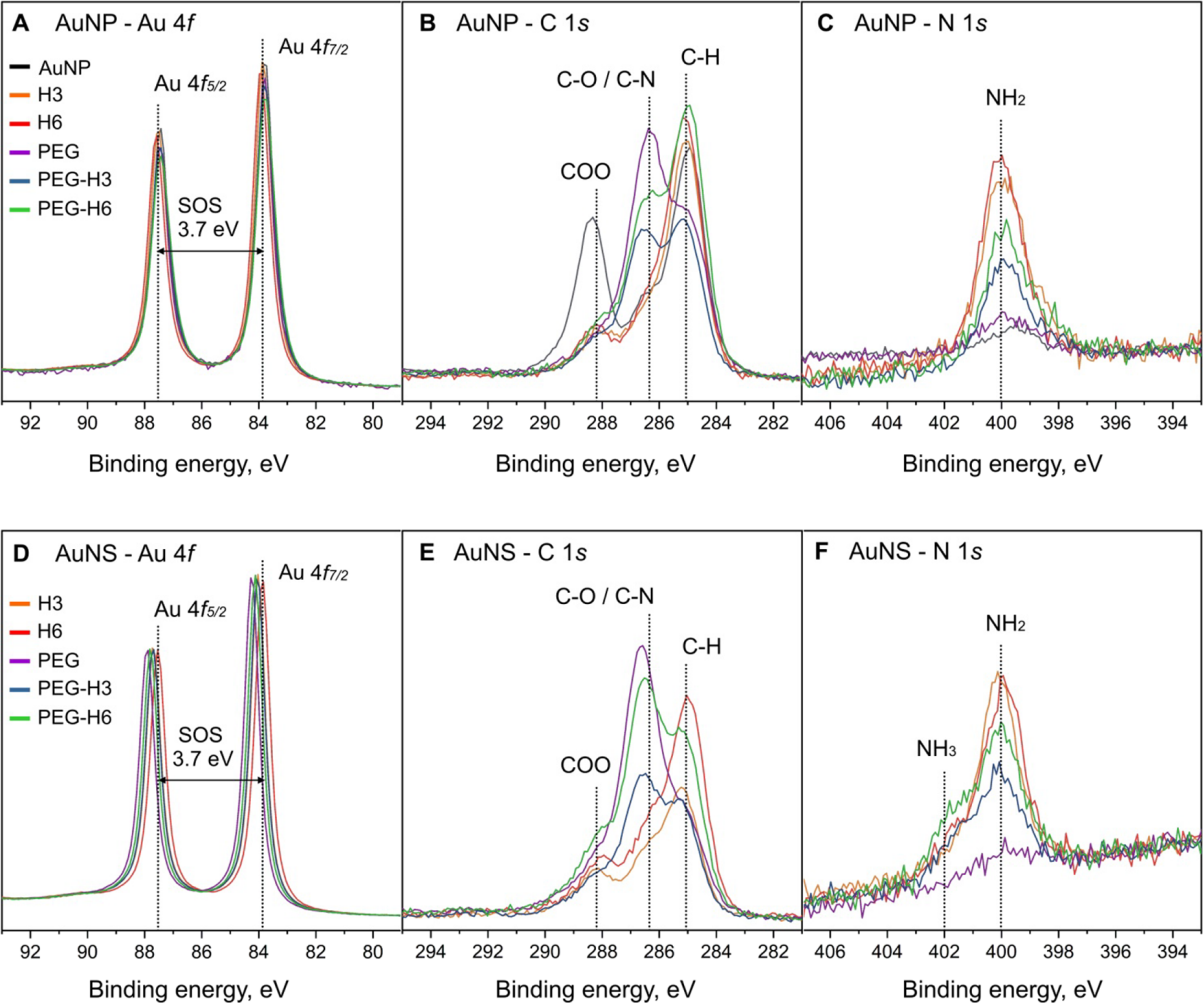
XPS core level spectra of AuNP (A–C, upper panel) and AuNS (D–F, lower panel) including Au 4f (A, D), C 1s (B, E), and N 1s (C, F). The spin orbit splitting (SOS) of the Au 4f core level is also included in panels (A) and (D).

We next used X-ray photoelectron spectroscopy (XPS) to quantify the presence of H3/H6 and PEG on AuNP/NS. All samples showed the signals expected from their synthesis as well as from the Si/SiO_2_ substrate used (Figures S3 and S4). The main core-level spectra of AuNP and AuNS are shown in Figure 3. The Au 4f core levels for both sample sets (Figure 3A,D) are typical for metallic gold, as expected from AuNP/NS. Small shifts in the binding energy (BE) on the order of 0.1 eV (AuNP) and 0.3 eV (AuNS) can be caused by either binding to H3/H6/PEG or be minor effects from sample charging. All spectra were aligned to the Si 2p_3/2_ core level of the substrate, which does not account for differential charging of the drop-cast nanostructures. The C and N 1s spectra were used to determine the presence of the peptides and the PEG through contributions specific to their intrinsic chemical states.

To aid identification of the chemical environments, reference measurements of H3, H6, and PEG were performed (Figure S3). Au nanostructures conjugated with H3/H6 showed three BE contributions to the C 1s core level (Figure 3B,E), typical for their amino acid constituents: 285.1 eV (aliphatic carbon) and 286.4/288.2 eV, which stem from the amine (N–H) and carboxyl (COO) functional groups, respectively. The BE for these chemical environments agree well with previous reference data of amino acid spectra.^35,36^ Samples with PEG showed an increased intensity around 286.4 eV associated with the C–O environments, which overlap in BE with the N–H feature from H3/H6. The N 1s core levels (Figure 3C,F) showed significant amounts of nitrogen only for samples including H3/H6 at a binding energy of 400.0 eV typical of the amine group (NH_2_). The unconjugated and PEG-only conjugated nanostructures only had trace amounts of nitrogen. In addition, the AuNS samples show an additional feature at higher BE (402.0 eV), which agrees with NH_3_ environments. Thus, the XPS data confirmed specific immobilization of H3, H6, and/or PEG on the nanostructures.

Quantitative ratios of AuNP/AuNS to peptide and PEG were determined from peak fit analysis to the respective core-level spectra (Table 1). Area ratios (Au 4f_7/2_ to N 1s core levels, Au:N) were used to determine the amount of surface-bound peptide in all samples. On average, H3/H6-AuNP/NS had Au:N = 2:1. In PEGylated samples, lower amounts of peptides were detected (Au:N between 2:1 and 3:1). From the ratios of the COO contribution from peptides and the C–O contribution from PEG to the C 1s core level, we determined ratios of peptide:PEG in the PEGylated samples (see the Supporting Information for details). PEG H6-AuNP/NS showed a slightly higher peptide load compared to PEG-H3 samples (Table 1).

**Table 1:** Quantification of the AuNP/NS and Functionalization Group Ratios from Peak Fit Analysis of XPS Core-Level Spectra^a^

sample	NP/NS:peptide	peptide:PEG
H3 AuNP	64.9:35.1 (1.85:1.00)	
H6 AuNP	64.5:35.5 (1.82:1.00)	
H3 PEG AuNP	75.7:24.3 (3.12:1.00)	26.8:73.2 (1.00:2.73)
H6 PEG AuNP	70.0:30.0 (2.33:1.00)	35.9:64.1 (1.00:1.79)
H3 AuNS	67.5:32.5 (2.08:1.00)	
H6 AuNS	64.9:35.1 (1.85:1.00)	
H3 PEG AuNS	74.5:25.5 (2.92:1.00)	30.5:69.5 (1.00:2.28)
H6 PEG AuNS	66.4:33.6 (1.98:1.00)	36.0:64.0 (1.00:1.78)

a The NP/NS:peptide ratio was determined from the peak areas of the Au 4f_7/2_ and N 1s core-level spectra. The peptide:PEG ratio is based on the contributions from COO groups from the peptides and C–O contributions from PEG to the overall C 1s core-level spectra. See Materials and Methods for details. Ratios are given in percent as well as relative to one (in brackets).

### Binding of H3/H6-AuNP/NS to Cultured N27 Cells and Hippocampal Neurons

**Figure 4:**
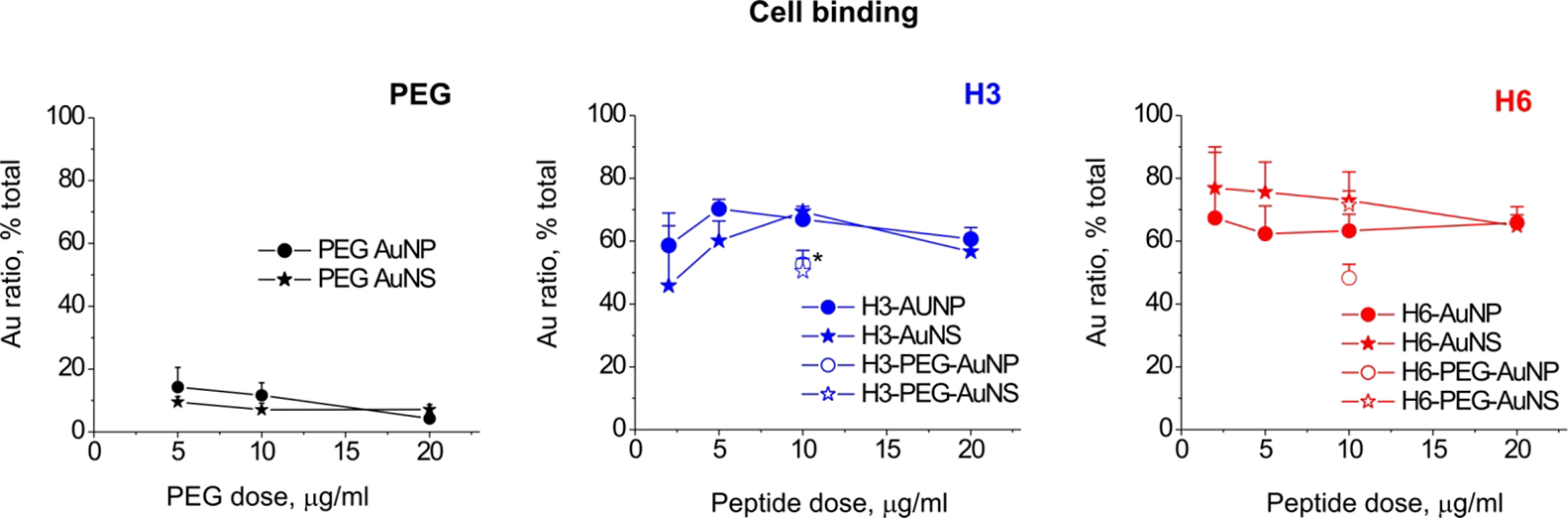
Binding of the +/–PEG-H3/H6-AuNP/NS to cultured N27 cells. In each graph, the ratio (%) of total gold content is detected in the cell-associated fraction for two nanostructure geometries. Three independent experiments. Results are expressed as means ± SEM. *H3-PEG-AuNS *vs* H3-AuNS; *p* = 0.051, H3-PEG-AuNP *vs* H3-AuNP, one-way ANOVA.

We next evaluated AuNP/NS binding to N27 cells, immortalized dopaminergic neurons. Cultured N27 cells (confluence 80%) were incubated with PEG- or H3/H6-AuNP/NS conjugates (2–20 μg/mL) for 24 h, and the cells were dislodged, spun down, and lysed. The Au content was measured in cell lysates using inductively coupled optical emission spectrometry (ICP-OES). Only a minor (5–10%) fraction of PEG only-conjugated AuNP/NS was bound to cells at any nanocompound concentration (Figure 4, PEG), whereas 40–80% of peptide-conjugated AuNP/NS was detected in the cell-associated fraction (Figure 4). PEGylated versions of the H3/H6-AuNP/NS at 10 μg/mL also interacted with N27 with binding ratios comparable to those of non-PEGylated versions (Figure 4, H3, H6), thus confirming that the H3/H6-functionalized nanocompounds efficiently bound to cell membranes. However, the PEGylated H3 formulations showed slightly lower cell-binding efficiency than non-PEGylated H3-AuNP/NS (Figure 4). The actual binding rate of the nanocompounds could be underestimated in our experimental design since cells were dislodged by trypsinization. Thus, the cell-associated fraction mostly contained membrane-embedded and internalized AuNP/NS; the nanostructures bound to cell surface receptors but not internalized or embedded were not expected to be present in this fraction.

**Figure 5:**
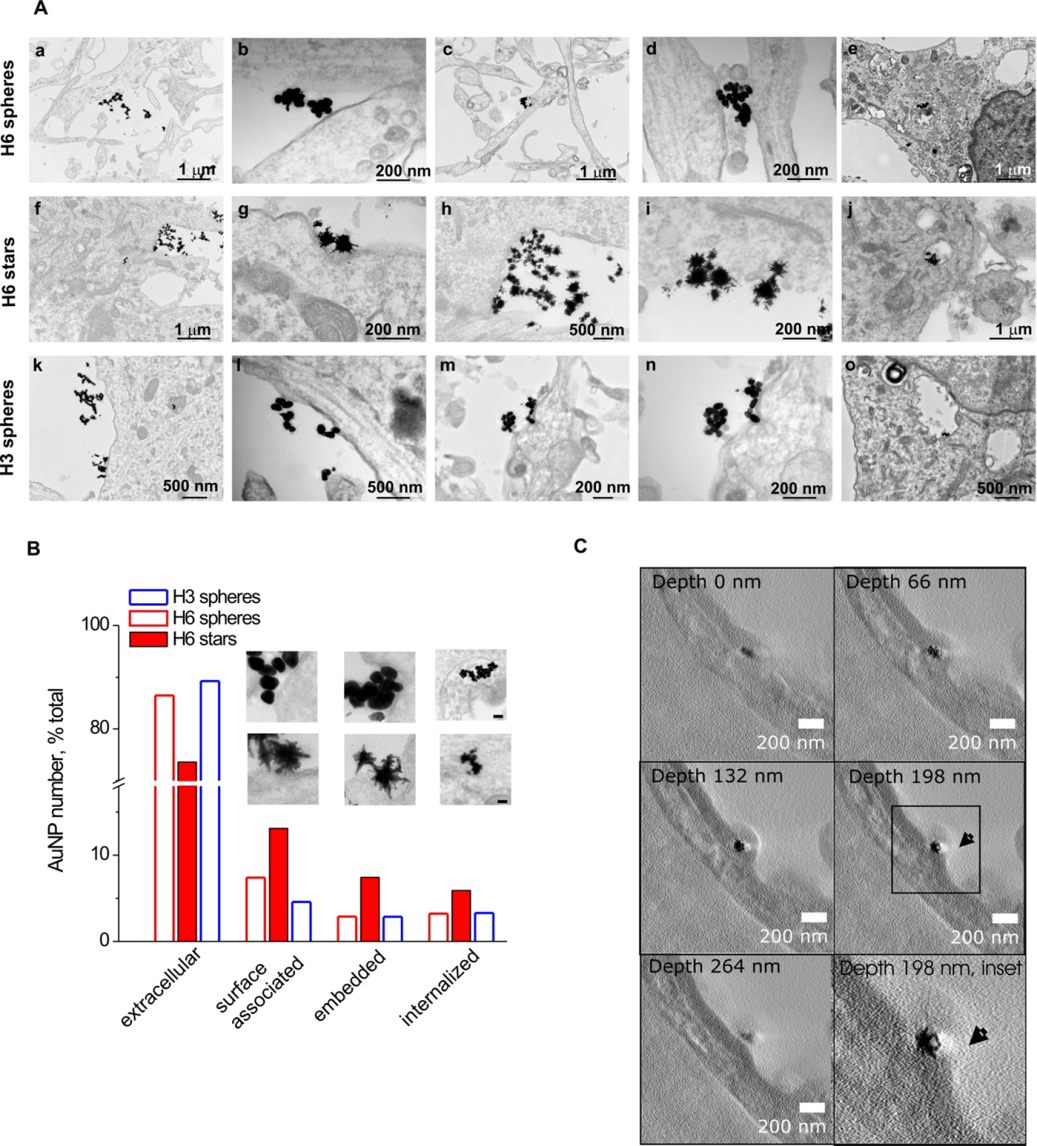
TEM showing interactions of H3 and H6 nanocompounds with cultured hippocampal neurons. (A) Individual and clustered H3/H6 nanoparticles (all 20 μg/mL, 24 h treatment) are predominantly detected adjacent to neurons and on cell surface (a,b,f,g,k,i,l) or embedded into (c,d,g,m,n) and/or piercing (g,h,i) the neuronal membranes. In rare cases, the aggregated nanoparticles were seen internalized in vesicle-like structures within neurons (e,j,o). (B) Quantification of AuNP distribution across cell compartments. See Materials and Methods for details. Total number of AuNP analyzed: H6 spheres, *n* = 1458; H6 stars, *n* = 984; H3 spheres, *n* = 1396. AuNP/NS counts in each compartment are normalized to the total AuNP/NS count for each particle type (set to 100%). (C) Orthoslices through the tomogram show an H3 nanostar embedded in the membrane of a neuron at a depth of 66 to 198 nm. The envelope opening can be seen at 198 nm depth (arrow) and is no longer seen at 264 nm.

We next examined the interaction of non-PEGylated S100 nanocompounds (20 μg/mL, 24 h treatment) with membranes of cultured hippocampal neurons using TEM. Individual and clustered H3- and H6-AuNP/AuNS were abundantly detected in the vicinity of neuronal plasma membranes (Figure 5A). They localized adjacent to neurons and on the cell surface (Figure 5Aa,b,f,k,i,l) or were embedded into (Figure 5Ac,d,g,m,n) and/or piercing (Figure 5A g,h,i) the neuronal membranes as both individual species and small aggregates. On single occasions, the AuNP/NS aggregates were internalized inside vesicular structures within neurons (Figure 5Ae,j,o). There was no significant difference between the cellular localization of H3- and H6-conjugated nanostructures.

Since our previous studies in non-neuronal cells showed that nanostars had a higher probability of binding to cell surface and/or internalization due to their multispiked geometry,^7^ we also tested this hypothesis in hippocampal neurons. To investigate the effect of AuNP/NS geometry on their (sub)cellular distribution, we focused on H6-conjugated compounds as H6 is a unique motif in the sequence of its parent protein, S100A4, and S100A4 has both extra- and intracellular receptor targets and, moreover, is internalized in several cell lines.^37^ For both geometries, we quantified the H6-AuNP/NS distribution across four compartments: *extracellular* (AuNP/NS not interacting with cell membranes), *surface-associated* (interacting with cell membranes without changes in membrane curvature, no embedding or piercing), *membrane-embedded* (embedded into and/or piercing cell membranes), or *internalized* (detected within cells). Since the samples represented sections of dissociated hippocampal cultures with low cell confluence (<10%), most H6-AuNP/NS were, as expected, detected extracellularly adhered to the polylysine substrate (Figure 5B) with more nanospheres than nanostars found in this compartment (86.5% vs 73.6%, respectively, Figure 5B, *extracellular*). However, the H6 nanostars were ca. 2 times more often associated with neuronal surface or internalized compared to H6 nanospheres (Figure 5B, *surface-associated* and *internalized*). Furthermore, the ratio of the membrane-embedded particles was approximately 2.5 times higher for H6 nanostars than for nanospheres (7.4% vs 2.9%, Figure 5B, *membrane-embedded*). The H3 nanospheres had a compartmental distribution similar to the H6 nanospheres (Figure 5B), thus supporting the hypothesis that the particle geometry *per se* can govern cellular distribution of nanocompounds.

Finally, to confirm that the AuNSs were able to embed into the plasma membrane of neurons, we employed 3D electron tomography of the H3-AuNS-stimulated sections (Figure 5C). Orthoslices through the representative tomography reconstruction of hippocampal neurons exposed to H3-AuNS illustrated that spikes of the AuNS appeared embedded within the plasma membrane (Figure 5C, depth of 198 nm, inset). The envelope opening can be seen at 198 nm depth (arrow) and is no longer present at 264 nm.

### S100-Derived Nanocompounds Induce Neurite Outgrowth from Cultured Hippocampal Neurons

**Figure 6:**
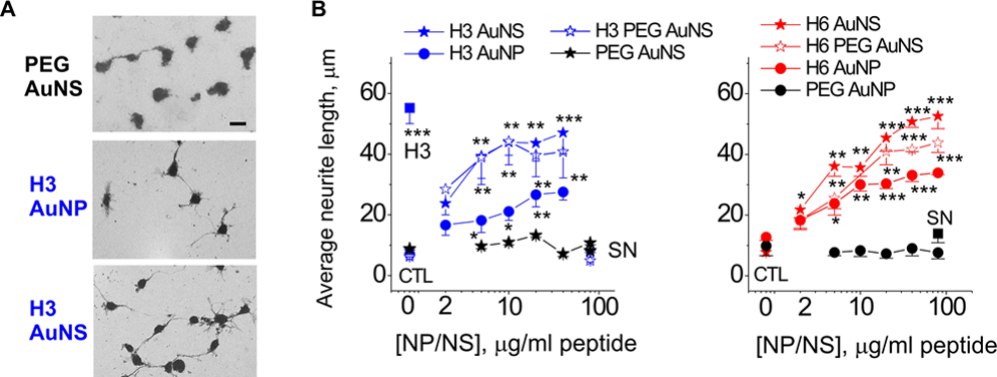
S100 peptide-functionalized AuNP/NS are neuritogenic in vitro. (A) Representative micrographs of hippocampal neurons treated with three different formulations of H3-conjugated nanocompounds (5 μg/mL). Scale bar, 10 μm. (B) H3- and H6-functionalized AuNP/NSs promote neurite outgrowth from hippocampal neurons. CTL, untreated cells; H3 – unconjugated peptide only (10 μg/mL); SN – centrifugation supernatants (no measurable peptide content, added to 10% of the total volume). One-way ANOVA *vs* CTL, four independent experiments. Results are expressed as means ±SEM. *p < 0.05; **p < 0.01; ***p < 0.001.

Since the S100A4 derivatives, the H3 and H6 peptides, are neuritogenic in vitro,^4−6^ we tested whether the H3/H6-functionalized nanocompounds can trigger neurite outgrowth from hippocampal cultures. Three nanoformulations of either peptide were tested: AuNS, PEG-AuNS, and spherical AuNP (Figure 6A,B). As a positive control, we used unconjugated H3 in its most effective concentration, 10 μg/mL (Figure 6B); as negative controls, either PEG only-conjugated AuNS or supernatants from the last AuNS centrifugation step (SN, Figure 6B) were employed.

All peptide nanocompounds induced neurite extension from hippocampal neurons (Figure 6), whereas the PEG-only conjugated AuNS did not. The potency or efficacy of H3- and H6-functionalized AuNS was not significantly affected by PEG conjugation. However, the nanostructure shape had a marked effect: both for H3- and H6-based nanocompounds, AuNSs were ca. 2 times more efficient as neurite inducers and also had higher potency (H3-AuNS *vs* H3-AuNP, 2.4 *vs* 4.0 μg/mL and H6 AuNS *vs* H6-AuNP, 4.3 *vs* 5.1 μg/mL, see Materials and Methods for the potency estimation) (Figure 6B).

Similar to (+/–PEG)-H3-AuNS, the (+/–PEG)-H6-AuNS promoted neurite extension, which was comparable with that induced by non-PEGylated compounds (Figure 6B). However, the H6-conjugated NP/NS demonstrated a higher degree of time-dependent aggregation in the stock solution and culture medium than H3-based formulations. Therefore, for neuroprotection and BBB crossing studies, we have focused on the H3-based nanocompounds.

### H3-Functionalized AuNP/NSs Are Neuroprotective in Cell Models of Oxidative Stress and PD

Unconjugated S100-derived peptides efficiently protect neurons in several in vitro models, including oxidative stress- and PD-related neurodegeneration.^4,6^ We therefore examined whether H3-AuNP/NSs were also neuroprotective in these assays.

**Figure 7:**
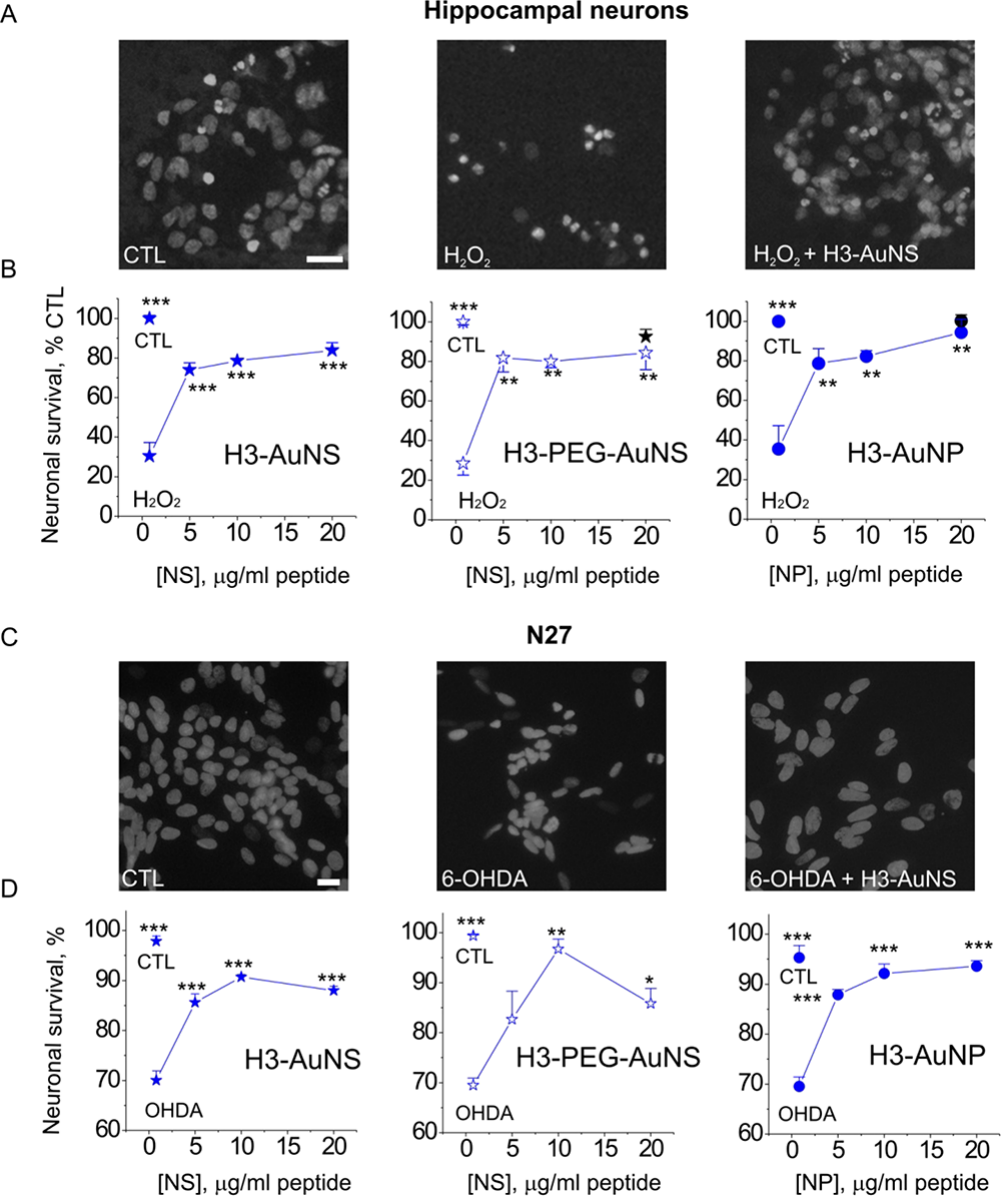
S100 peptide-based AuNPs are neuroprotective in vitro. (A,C) Representative images of Hoechst-stained hippocampal neurons (A) or N27 cells (C) left untreated (CTL) or treated with H_2_O_2_/6-OHDA (A or C, respectively) in the absence or presence of H3-AuNS (10 μg/mL). Scale bar, 20 μm. (B,D) H3-functionalized AuNP/NS protect cultured hippocampal neurons and N27 cells against the H_2_O_2_- (B) and 6-OHDA-induced (D) neurotoxicity. CTL, untreated cells; H_2_O_2_/6-OHDA, neurotoxin only-treated (60 and 100 μM, respectively) cells. Naked AuNP or PEG-AuNS (B, black filled circles/stars) does not affect basal neuronal survival. One-way ANOVA vs H_2_O_2_ (B) or 6-OHDA (D), three to four independent experiments, *p < 0.05; **p < 0.01; ***p < 0.001.

The nanocompounds were first tested in a neuronal model of oxidative stress, which is a key neurodegeneration factor in several brain disorders.^38,39^ As a model neurotoxin, we used hydrogen peroxide (H_2_O_2_), an oxidant commonly employed in oxidative stress models^39−41^ and in our previous reports shown to induce neurodegeneration in multiple cell types.^4,6^ H_2_O_2_ treatment profoundly decreased neuronal viability (ca. 4-fold, H_2_O_2_, Figure 7A,B). All three H3 nanocompounds efficiently protected neurons from H_2_O_2_-induced death in a dose-dependent manner, with the potency of the H3-AuNS being higher than that of the spherical H3-AuNP (1.7 *vs* 4.5 μg/mL peptide, see Materials and Methods for details).

We have previously shown that H3 and H6, as well as their parent protein, S100A4, induce neurite extension in dopaminergic neurons^6,42^ and protect them from cytotoxicity induced by the model PD neurotoxin, 6*-*hydroxydopamine (6-OHDA).^6^ 6-OHDA evokes several pathological hallmarks of PD in animals: dopamine depletion, selective degeneration of DA neurons, neurological deficits,^43^ and triggers cell death in cultured primary and immortalized DA neurons (N27 cells).^6,44−46^ Similar to dopaminergic neurons, N27 cells are positive for DA marker tyrosine hydroxylase (TH) and dopamine transporters^47,48^ and are widely used as an in vitro PD model.^49−51^

In agreement with previous studies, 6-OHDA decreased viability of N27 cells by ca. 30% in our experimental setup (OHDA, Figure 7C,D), and all three H3 nanoformulations had a significant pro-survival effect in this model. There was no significant difference in either potencies or efficacies of the three tested compounds (Figure 7D).

### H3, H6, And Their Nanoderivatives Decrease ROS Generation Following Oxidative Stress in Hippocampal Neurons

**Figure 8:**
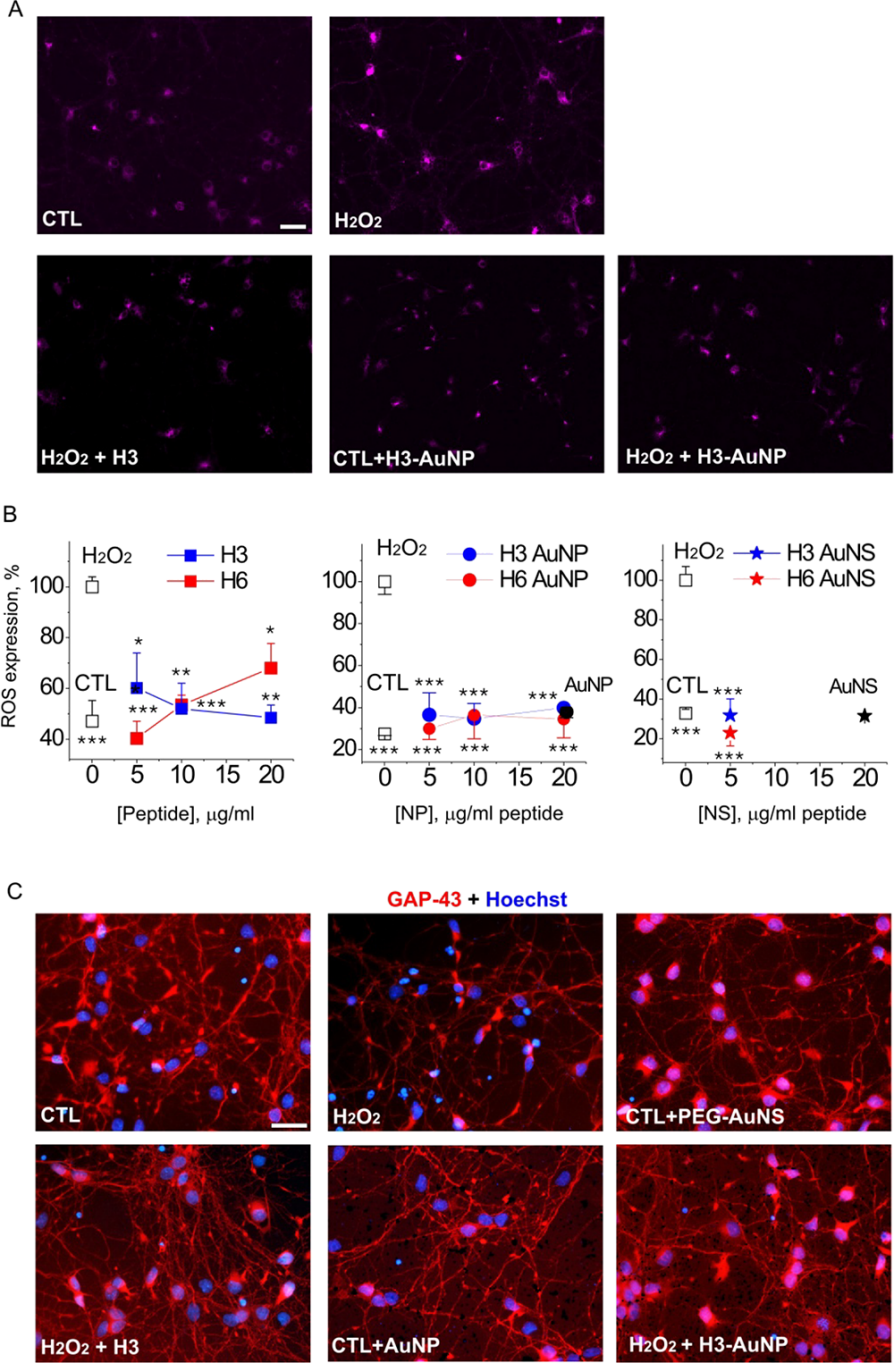
H3- and H6-functionalized AuNP/NS decrease ROS generation following oxidative stress. (A) Representative images of ROS staining of hippocampal neurons left untreated (CTL), treated with H3-AuNP only (CTL + H3-AuNP), or treated with H_2_O_2_ in the absence or presence of H3 (10 μg/mL) or of H3-AuNP (10 μg/mL peptide). Scale bar, 20 μm. (B) H3, H6, and the peptide-functionalized AuNP/NS reduce ROS levels in the H_2_O_2_-treated hippocampal neurons. CTL, untreated cells; H_2_O_2_, neurotoxin only (60 μM)-treated cells. AuNP/AuNS, negative control (filled circles/stars, PEG only-functionalized nanostructures). One-way ANOVA *vs* H_2_O_2_, three to four independent experiments, *p < 0.05; **p < 0.01. (C) Representative micrographs of hippocampal neurons left untreated (CTL) or treated with H_2_O_2_, H3, naked, or H3-conjugated AuNP/NS (20 μg/mL peptide) in the indicated combinations for 24 h. Double staining for GAP-43 to visualize neuronal morphology and Hoechst to detect pyknotic nuclei. Scale bar, 20 μm.

Reactive oxygen species (ROS) are produced during several neurological disorders, including PD and AD, as a result of mitochondrial/genetic dysfunction, neuroinflammation, and impaired cellular homeostasis,^52^ and prominently contribute to neurodegeneration. In our model, challenging hippocampal neurons with extracellular H_2_O_2_ resulted in a notable increase in ROS generation after 24 h (Figure 8), which was strongly diminished by the peptides alone in a dose-dependent mode and also by the H3/H6-AuNP/AuNS. Nanocompounds that had not been functionalized with the peptides did not affect ROS generation (Figure 8B). Thus, H3/H6 and their nanoderivatives could robustly counteract the rise of the intracellular ROS levels known to cause damage to lipids, proteins, and DNA in neurological disorders.^53^ Importantly, this, to our knowledge, is the first demonstration that neuritogenic motifs of the S100A4 protein can suppress ROS production, suggesting that the parent protein may also be able to modulate neuronal ROS levels.

### H3-AuNPs Protect Cultured Hippocampal Neurons from Toxicity Induced by Oligomeric Amyloid β (Aβ)

Our previous data^6^ suggests that S100A4 and its peptide derivatives (H3/H6) interact with and exert neuroprotection *via* ErbB family receptors and prevent the Aβ-induced disruption of the neuronal membrane structure*.* Thus, they may launch intracellular pro-survival pathways and/or compete with the deleterious interactions of the Aβ with ErbBs and the membrane bilayer.^27^ However, the effect of S100 derivatives or nanocompounds on neuronal survival in the in vitro AD model has not been studied.

**Figure 9:**
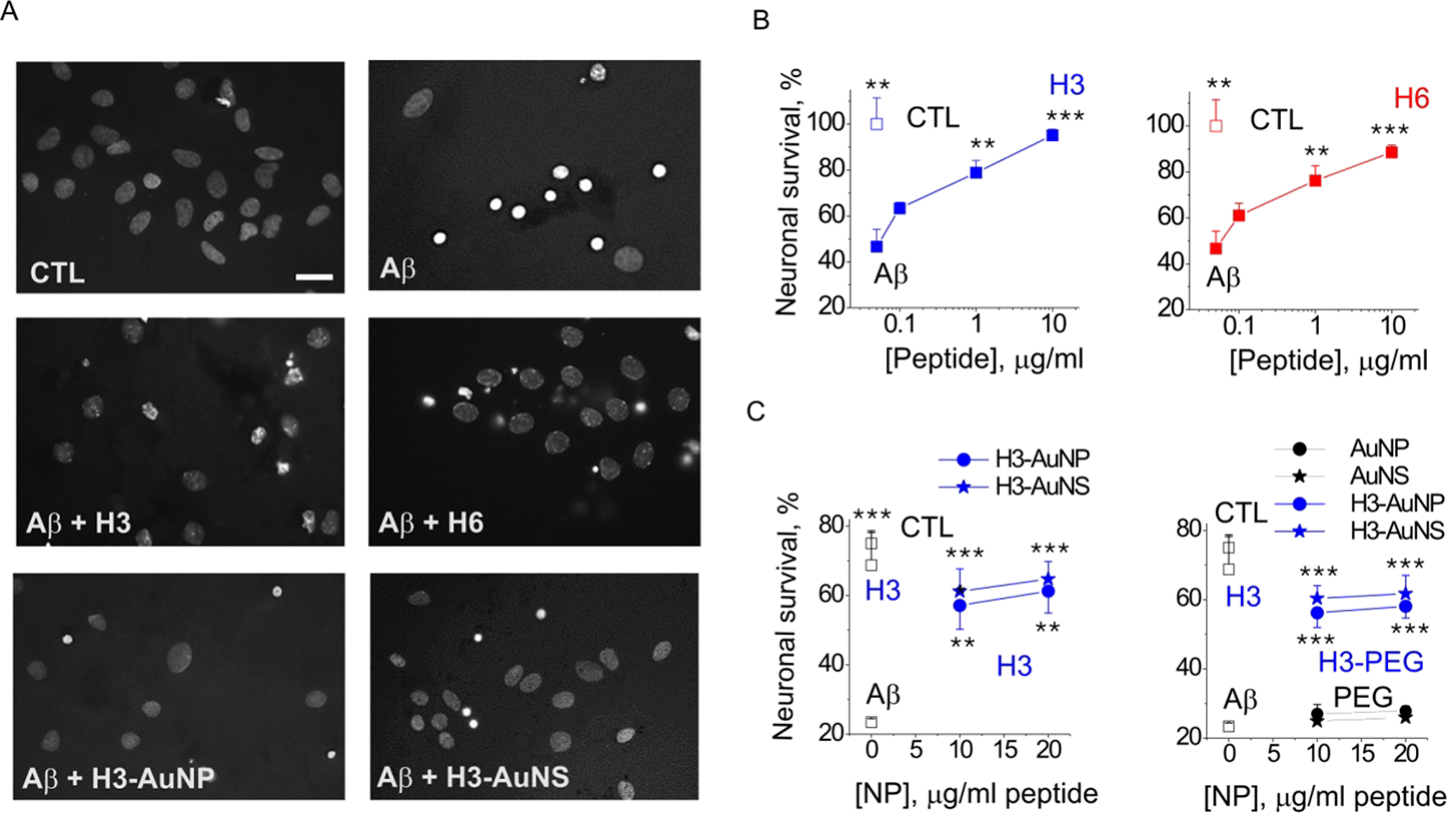
S100A4 mimetic peptides and peptide-conjugated AuNP/NSs protect hippocampal neurons against Aβ-induced neurotoxicity in vitro. (A) Representative images of Hoechst-stained hippocampal neurons left untreated (CTL) or challenged with oligomeric Aβ_1-42_ (Aβ) in the absence or presence of H3/H6 peptides, H3-AuNP (nanospheres), or H3-AuNS (nanostars, all 10 μg/mL). Scale bar, 10 μm. (B,C) H3 and H6 peptides (B) and (+/–PEG)-H3-functionalized AuNP/NSs (C) protect cultured hippocampal neurons against Aβ-induced death. CTL, untreated cells; Aβ (8 μM) – neurotoxin only-treated cells. H3-PEG, H3 and PEG-functionalized AuNP/NS. PEG, PEG-only conjugated AuNP/NS. One-way ANOVA vs Aβ, three to four independent experiments, *p < 0.05; **p < 0.01, ***p < 0.01.

We therefore evaluated the neuroprotective effect of H3, H6, and H3+/-PEG-AuNP/NS in hippocampal neurons subjected to oligomeric Aβ. The Aβ_1-42_ treatment strongly (ca. 2.5-fold) reduced neuronal viability after 48 h (Figure 9A–C), and this decrease was counteracted by both H3 and H6 peptides in a dose-dependent manner (Figure 9B). H3 nanoformulations, both +/–PEG, robustly increased survival of Aβ-challenged neurons (Figure 9C) with no significant differences in the neuroprotective effect between nanocompound geometries. PEG-only conjugated AuNP/NS did not affect the death rate of Aβ-treated neurons (Figure 9C, right, PEG). Thus, both unconjugated S100-derived peptides and the +/–PEG H3 nanocompounds were efficient in the neuronal model of AD.

### S100 Nanomaterials Cross a Model Blood–Brain Barrier and Are Not Toxic In Vivo

**Figure 10:**
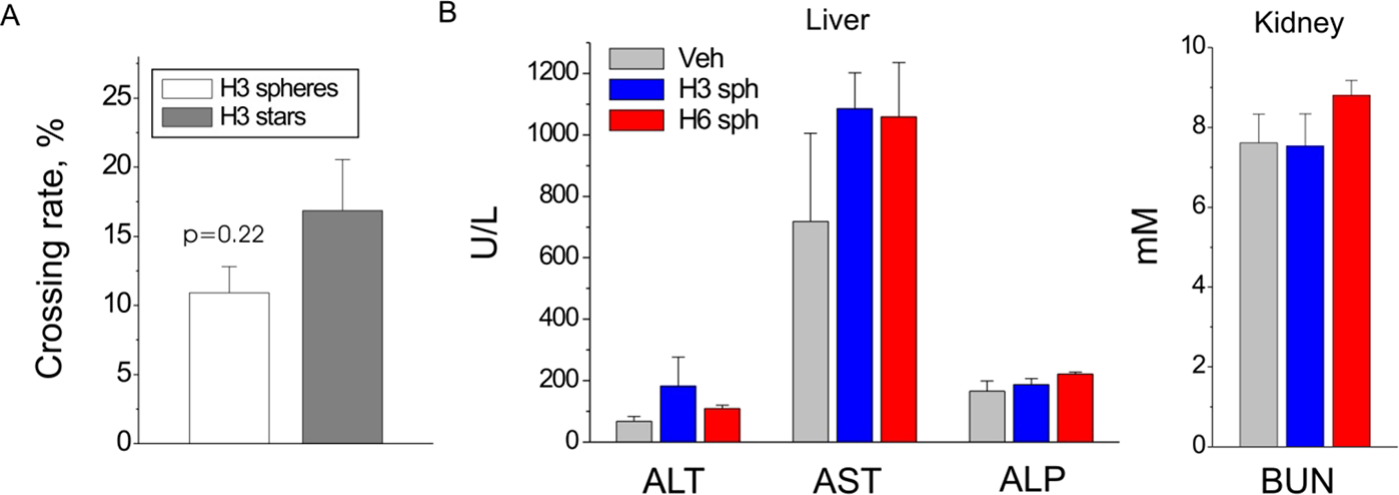
Peptide-conjugated nanostructures cross a model BBB and do not affect liver and kidney function in vivo. (A) Crossing rates of H3-conjugated nanospheres and nanostars in a triple-co-culture Transwell BBB model, 48 h, number of inserts *n* = 3/3 (spheres/stars), two-tailed *t* test. (B) Blood plasma levels of markers of liver and kidney injury in mice treated with the vehicle (Veh), H3-nanospheres (H3sph, 5 mg/kg), or H6-nanospheres (H6sph, 5 mg/kg) for 24 h. ALT, alanine aminotransferase; AST, aspartate aminotransferase; ALP, alkaline phosphatase; BUN, blood urea nitrogen. No statistically significant difference was found between the groups, one-way ANOVA vs Veh, number of animals *n* = 5/5/5 (Veh/H3 spheres/H6 spheres).

Our previous studies have shown that AuNS can cross the plasma membrane of brain endothelial cells in high amounts (ca. 20% efficiency of BBB transport in vitro^7^). We tested H3-AuNP/NS in a three component co-culture Transwell BBB model comprising human brain endothelial cells (HCMEC/D3), pericytes (HBVPs), and astrocytes. This model more closely resembles the in vivo BBB compared to one-component BBB models (ref (54) and references therein). This three-component system has also been shown to have greater barrier strength than single HCMEC models^54^ and has been used to study drug penetration through the BBB in vitro^55^ and the modulation of the BBB function by antioxidants during experimental hyperglycaemia.^56^ A schematic representation of a triple-co-culture model of the BBB used in our study is shown in Figure S5. The integrity of the BBB was assessed by measurement of its permeability to dextran-rhodamine B (70 kDA). The calculated apparent permeability coefficients for dextran (Figure S5c) were comparable with previous BBB studies using Transwell models.^57,58^ Thereafter, H3-conjugated spheres or stars were added to the corresponding insert and the plates were incubated for 48 h. Aliquots of media from the inserts and from the wells were collected for UV–vis measurements, and H3-AuNP/NS BBB crossing rates were calculated (Materials and Methods). Both the H3-AuNP and H3-AuNS crossed the model BBB with efficiencies of 10.9 ± 1.9% and 16.9 ± 3.7% (mean ± SEM), respectively (Figure 10A). The rate of H3-AuNS crossing was comparable with our previously published data^7^ and higher than that of the H3-AuNP but this trend did not reach statistical significance (*p* = 0.22).

We then tested whether H3- or H6-conjugated nanomaterials were toxic in vivo. Healthy mice were treated with either a saline vehicle or with H3 or H6 nanospheres (*D* ≈ 50 nm), and blood biochemistry was analyzed for the clinical markers of liver or kidney injury. In our previous studies, unconjugated H3 or H6 was cytoprotective and did not cause detectable side effects in vivo*.*^4−6^ Accordingly, H3- or H6-conjugated nanospheres did not significantly affect plasma levels of liver or kidney injury markers (Figure 10B), thus confirming that our nanocompounds were not toxic in vivo.

## Discussion

Here, we designed an effective neuroprotective platform combining multifunctional S100A4-derived dendrimers H3 and H6 with nanostars/spheres that can be further surface-functionalized to increase BBB crossing and cell targeting.

NSs can potentially offer several benefits over spherical NPs in (neuro)theranostics. First, the multispiked morphology of nanostars provides an easy access to target molecules on cell membranes and a large supporting surface to which neuroactive agents and sensors can adsorb. In this study, both spherical and star-shaped H3-AuNPs were neurotrophic in cultured neurons and the neuritogenic effect of the nanostars was markedly higher (Figure 6). The H3/H6-AuNS had a potency and efficacy similar to those of unconjugated peptides and was 1.5–2 times more efficient than its spherical counterparts. This could be explained by the higher measured AuNS binding to/embedding into the neuronal surface (Figure 5) compared to the spheres, resulting in strong activation of neuritogenic signaling and robust neurite adhesion to the substrate. Additionally, interaction of the AuNS with plasma membranes is known to induce a slight increase of neuronal electrical activity^59^ and thus local Ca^2+^ ‘hotspots’, which can promote neurite extension.

The H3-AuNP/NS also protected neurons against the oxidant- or PD toxin (6-OHDA)-induced cytotoxicity (Figure 7), with the H3-AuNS having the highest potency in the oxidative stress model. Similarly, both unconjugated H3 or H6 and the H3 nanoconjugates improved survival of hippocampal cultures subjected to the AD toxin Aβ (Figure 8). These results align with our previous data showing that H3 and H6 peptides launch multiple neuroprotective pathways by activating plasma membrane receptors such as ErbB^6^ and/or preventing toxin-induced plasma membrane disruption (Aβ).^27,28^ This multifactorial neuroprotection may be an important advantage for future translational studies providing therapeutic effects for a broad spectrum of pathological conditions. ErbBs are known to be overexpressed at lesion sites in AD,^60^ and ErbB activation improves neuronal survival and synaptic function in AD models.^17,24^ Furthermore, given that some S100 proteins are associated with Aβ plaques and can affect formation of Aβ aggregates,^61^ it is possible that H3 and/or H6, as well as S100A4 itself, may interact with Aβ and exhibit similar properties, presenting a fascinating field of future research. Importantly, the finding that peptides encompassing S100A4 motifs decreased the AD-related neurodegeneration for the first time involves S100A4 in AD development.

Our nanomaterials did not affect markers of liver and kidney damage in vivo, confirming their biocompatibility. PEGylation did not significantly alter the efficiency of our compounds adding a component to increase the half-life and circulation time of our hybrids in vivo, where it is known to decrease NP phagocytosis and non-specific distribution.^62^ PEG has been approved by the FDA as safe and biodegradable;^63^ moreover, PEGylation reduces systemic NP toxicity as well as nanoneurotoxicity,^64^ and several PEGylated NPs are currently used in humans as drug carriers for actively targeted cancer therapy (ref (63) and references therein).

Interestingly, some features of the neuroprotective and anti-oxidative effects of our nanocompounds were similar to those of other promising biocompatible nanomaterials, tetrahedral framework nucleic acids (tFNAs), whose beneficial effects in a model stroke are mediated by downregulating the Toll-like receptor signaling pathway and activating PI3K-AKT (refs (65) and (66) and references therein). This suggests that flexible nanoplatforms targeted at several neuroprotective mechanisms can be a basis for efficient multipurpose neuroprotectants.

A potential strength of using nanostars as drug delivery vehicles is their ability to cross the BBB. Recently, we have shown that the AuNS conjugated with a non-targeting ligand 4-ethylcatechol can cross the plasma membrane of brain endothelial cells in high amounts (ca. 20% efficiency of BBB transport in vitro^7^) and can be functionalized with BBB-targeting molecules almost doubling the transport efficiency (up to 40%).^7^ Accordingly, other studies show that nanoparticles can cross the BBB by multiple mechanisms (refs (67−69) and references therein). In this study, H3-conjugated nanostars crossed a triple-co-culture model of the BBB at ca. 17% efficiency, demonstrating the potential of nanostars as drug carriers. Interestingly, functionalization with H3 could also contribute to the ability of the nanocompounds to cross the model BBB. S100A4 is known to bind receptor for advanced glycation end products, RAGE (ref (4) and references therein), with the H3 motif reportedly involved in binding. In studies from other groups, RAGE-interacting compounds efficiently crossed the BBB and robustly increased the brain delivery of AuNPs by a RAGE-mediated transcytosis.^70,71^ RAGE is also upregulated in AD and serves as an ‘influx receptor’ for Aβ (ref (72) and references therein), suggesting that S100A4 compounds may have increased efficiency under AD conditions. Optimization of our peptide nanomaterials as the ‘BBB shuttles’ is a promising area for future research and drug development.

Gold nanostars are also plasmonic and absorb near infrared (NIR) light for photothermal therapy^73^ and can, in future, also be used for bioimaging employing label-free, non-invasive X-ray computed tomography^74^ or fluorescence, making them candidates for both therapy and imaging in the clinic. Finally, due to its modular structure, our platform can be easily modified, with, e.g., Au substituted for FDA-approved materials that degrade into non-toxic products, and can pave the way for future preclinical studies. Based on the design of our H3/H6 gold nanostructures, these biodegradable nanoparticles should be promising candidates for translation into therapy.

In conclusion, our results indicate an advantage of using nanostar drug delivery vehicles over regularly shaped spherical NPs to improve the potency and efficacy of cargo drugs and decrease effective drug doses. These effects may be more pronounced for physiological processes involving a ’mechanical’ component, such as plasma membrane remodeling or changes in cell adhesion during neurite outgrowth. In combination with our neurotrophic peptides, nanostars hold promise as a theranostic platform with multiple orthogonal functions, such as neuroprotection, and delivery of high drug loads across the BBB. Additional applications may include photothermal therapy (AuNP) and bioimaging. Since our peptides are neurotrophic in several neuron types, we expect them to be effective in treating multiple neurodegenerative conditions, including oxidative stress, excitotoxicity, PD, and AD. An important feature of our nanoplatform is use of multibranched peptide dendrimers, which increases the compound potency and allows functionalization without affecting bioactive motifs. Thus, our peptide-NS platform has the potential both as a versatile neuroprotectant and as technology to improve the efficiency for small molecule drugs that cannot cross the BBB, which may improve treatment of a broad spectrum of neurological diseases.

## Materials and Methods

### Materials and Peptides

Tetraethylorthosilicate (TEOS), cetyltrimethylammonium bromide (CTAB), sodium hydroxide (NaOH), chloroauric Acid (HAuCl_4_), silver nitrate (AgNO_3_), hydrochloric acid (HCl), sodium citrate dibasic trihydrate, gold chloride trihydrate (HAuCl_4_·3H_2_O), sodium citrate tribasic dihydrate, silver nitrate (AgNO_3_), l-ascorbic acid (AA), hydrogen peroxide solution (H_2_O_2_, 30 wt %), (3-mercaptopropyl)trimethoxysilane (MPTMS, 95%), phosphate-buffered saline (PBS, pH 7.4), DMEM tissue culture medium, and bovine serum albumin (BSA) were purchased from Sigma-Aldrich, UK. Hydrochloric acid (HCl, 37%), sulfuric acid (H_2_SO_4_, 96%), acetone, and 2-propanol were obtained from VWR International, UK. Deionized (DI) water was purified using the Millipore Milli-Q gradient system (>18.2 MΩ).

Peptides (sequences H3: KELLTRELPSFLGKRT, H6: NEFFEGFPDKQPRKK) were synthesized as tetramers composed of four monomers coupled to a lysine backbone (Schafer-N, Denmark). Tetramerization was previously found to be necessary for the neuritogenic activity of S100A4.^23^

### Gold Nanostar Synthesis and Peptide Conjugation

The synthesis scheme of the nanostructure preparation (+/–PEG-H3/H6-AuNP/NS) is shown in Figure 1. Gold nanostars were synthesized using a surfactant-free seed-mediated method,^75^ as reported in our previous work.^3^

Briefly, spherical citrate-stabilized Au nanoparticle (AuNP) seeds, with average sizes of 12 nm, were prepared by heating 100 mL of a 0.25 mM aqueous HAuCl_4_·3H_2_O solution in a 250 mL Erlenmeyer flask, under magnetic stirring. Once the solution reached the boiling point, 1 mL or 0.25 mL (for AuNP 50 nm) of a 3% (w/v) aqueous sodium citrate solution was rapidly added, under vigorous stirring. Color started appearing in the solution after 1–3 min, and heating was continued until it became a stable bright red color (within 10 min). The solution was then cooled in an ice bath and washed by centrifugation, its volume was made up to 100 mL with Milli-Q water, and it was stored at 4 °C. Then, 100 μL of 3 mM AgNO_3_ and 50 μL of 100 mM ascorbic acid were quickly added. The color of the solution changed from faint red to blue-green as soon as AA was added, and stirring was stopped after 30 s. For AuNS synthesis, 200 μL of the 12 nm Au seeds was added to 10 mL of 0.1 mM HAuCl_4_·3H_2_O with 10 μL of 1 M HCl at RT, under moderate stirring. Then, 100 μL of 3 mM AgNO_3_ and 50 μL of 100 mM ascorbic acid were quickly added. To fabricate PEG-AuNS, 125 μL of 1 mM polyethylene glycol (PEG) was added after completion of synthesis. To fabricate the +/–PEG-H3/H6-AuNP/NS, the H3 or H6 peptide was added either alone or with the peptide:PEG volume ratios as shown in Table 2 and incubated at RT while stirring for 24 h. +/–PEG-H3/H6-AuNP was prepared using the 50 nm Au nanospheres with the peptide/PEG ratios as shown in Table 2. Following conjugation, all nanostructures were washed by centrifugation–resuspension three times and final peptide concentrations in the obtained suspensions were quantified using a Nanodrop (ThermoFisher Scientific, UK). Thereafter, nanostructures were aliquoted and stored in the dark at 4 °C until use (1–14 days after the preparation). For in vivo studies, commercially available AuNPs (50 nm, Sigma-Aldrich) were used. For biological studies, nanostructures were sonicated (30 s) before use. For the particle stability studies, the H3- or H6-conjugated nanospheres (100 μL) were pelleted (8000 rpm × 10 min), and the peptide concentration in supernatants was measured using a Nanodrop and expressed as a percentage of the initial peptide concentration in the nanoparticle suspension. The measurements were performed on days 0, 5, 11, and 18 after the nanoparticle preparation.

**Table 2:** Peptide:PEG Ratios for Gold Nanostars (AuNS, Left Panel) and Nanospheres AuNP (Right Panel)

conjugation	peptide volume, 2 mg/mL (μL)	PEG volume, 1 mM (μL)	conjugation	peptide volume, 2 mg/mL (μL)	PEG volume, 1 mM (μL)
AuNS PEG		125	AuNP PEG		250
AuNS H3	50		AuNP H3	100	
AuNS H6	50		AuNP H6	100	
AuNS H3 + PEG	37.5	31.2	AuNP H3 + PEG	75	62
AuNS H6 + PEG	37.5	31.2	AuNP H6 + PEG	75	62

Zeta (ζ) potentials of the coated nanocompounds were measured in RPMI cell growth medium (RPMI), neurobasal medium (NB), and 1% BSA. For each condition, 40 μg/mL (peptide concentration) of the respective nanocompound was added to 1 mL of the medium and ζ potentials were measured using the Zeta sizer Nano (Malvern instrument 2000) at RT. Size distributions of the naked and H3-conjugated nanospheres in deionized water were measured by DLS (Zeta sizer Nano). Each measurement was performed in triplicate, with the arithmetic mean reported.

### Transmission Electron Microscopy (TEM)

The morphology of (+/–PEG)-H3/H6-AuNP/NS was examined by TEM using Holey carbon 300 Cu TEM grids. High-resolution TEM was performed using a Jeol 2100 TEM operated at 200 kV. The AuNP ellipticity was calculated from TEM images, defined as the ratio of the average major axis/average minor axis of AuNPs obtained by Gaussian fitting of the particle size distributions (*n* = 62). The average sphericity indices (SI) of AuNP/AuNS (*n* = 62/31) were calculated as SI = 4π*S*/*P*^2^, where *S* is the TEM projection area and *P* is a perimeter of a given nanosphere/nanostar, as previously described.^76^

To study AuNP interaction with neurons, 7 DIV cultures of dissociated hippocampal neurons grown on poly-l-lysine coated Thermanox coverslips (Thermo Fisher, UK) were treated with selected nanocompounds (H3/H6 nanospheres or nanostars) for 24 h, rinsed with 0.1 M 4-(2-hydroxyethyl)-1-piperazineethanesulfonic acid (HEPES) buffer (pH 7.2), fixed with 2.5% glutaraldehyde in HEPES for 2 h at 4 °C, and washed 3 × 10 min with HEPES. The samples were then bulk-stained with osmium tetroxide (1% OsO_4_ in HEPES × 1 h at RT) to enhance contrast from cellular organelles. After washing six times with deionized (di) H_2_O, cells were dehydrated in a graded ethanol concentration (50%, 70%, 95%, and dry 100%, volume ratio of ethanol to diH_2_O), 3 × 5 min followed by 3 × 10 min in acetonitrile (Sigma-Aldrich, UK). The samples were then progressively infiltrated with 50%, 75%, and 100% solutions of Taab 812 resin (Sigma) in acetonitrile at RT (2 h × 50% resin, overnight × 75% resin, and 3 × changes of 100% resin over 3 days) and cured at 60 °C for 24 h. The embedded neurons were cut using a Leica UC7 ultramicrotome (Leica, Austria) with a 35° diamond knife (Diatome, CH) to a thickness of 70 nm for TEM and 400 nm for STEM. Sections were immediately collected on 2 × 1 mm slot grid Formvar and carbon-coated TEM copper grids (Agar Scientific, UK), dried, and stored until TEM analysis. TEM imaging of hippocampal neurons was done at 120 kV (JEOL JEM 1400Plus, JEOL, Japan) with a 2*k* by 2*k* format CCD camera (JEOL Ruby CCD Camera, JEOL, Japan). Tilt series were collected at 200 kV (JEOL JEM F200 JEOL, Japan) with the Recorder software in STEM mode using the bright-field imaging detector (JEOL, Japan) from −60 to +60°, with 1° increments.

To quantify AuNP/NS interaction with neuronal membranes, a total of 28 (H3 nanosphere, H6 nanostar) or 32 (H6 nanosphere) non-overlapping TEM images from multiple neurons were generated and examined for localization of each AuNP/NS, with the total number of AuNP/NS analyzed *n* = 1000–1500 for each particle type. Nanostructures were assigned to four compartments: *extracellular* (not interacting with cell membranes), *surface-associated* (interacting with cell membranes without changes in membrane curvature, no embedding or piercing), *membrane-embedded* (embedded into and/or piercing cell membranes), and *internalized* (detected within cells). AuNP/NS counts in each compartment were normalized to the total count *(extracellular + surface-associated + membrane-embedded + internalized)* for each particle type (set to 100%).

Image alignment and tomographic reconstruction using the weighted back-projection reconstruction technique was performed using Etomo (part of IMOD Version 4.11).^77,78^ Visualization of the three-dimensional reconstruction was performed using Avizo software (v9.1, FEI, USA).

### X-ray Photoelectron Spectroscopy (XPS)

Gold nanoparticles and their binding to PEG and H3/H6 were investigated using X-ray photoelectron spectroscopy (XPS). Survey and core-level spectra were recorded on a K-Alpha+ XPS system (Thermo Fisher) using its monochromated, microfocused Al Kα X-ray source (*h*ν = 1486.7 eV) with a base pressure of 2 × 10^–9^ mbar and X-ray spot size of 400 μm. Data were collected at 200 eV pass energy for survey and 20 eV pass energy for core-level spectra. The nanoparticles were drop-cast onto Si wafer substrates (Si/SiO_2_), which were mounted on the sample holder using conducting carbon tape. A flood gun was used to minimize sample charging throughout. The data were analyzed using the Avantage software package (Thermo Fisher), and for peak fit analysis, Voigt functions with Shirley-type backgrounds were used.

The ratios of the peptides to the PEG were determined based on peak fits of the C 1s core levels. The areas of the COO contribution and the overlapping C–O/C–N contribution were extracted. To remove the contribution of C–N states from the C–O contribution specific to PEG, a ratio of 1:1 was assumed between COO and C–N and the equivalent area was subtracted. The resulting area ratios were then corrected for the number of environments for each peptide/PEG. The PEG used had an *M*_W_ of 2100 Da, with 47 PEG subunits each containing two C–O environments and two C–O end groups, yielding a total of 94 C–O carbons. Both H3 and H6 were used in a tetramer form containing 16 and 15 amino acids for each strand, respectively. The connecting entity of the tetramer is formed of 5 further amino acids. This gives a total of 69 and 64 COO environments for H3 and H6, respectively. The peak areas were corrected for this unequal ratio of functional C environments, and the resulting ratios were scaled to 100%.

### Cell Lines and Animals

The rat immortalized mesencephalic dopaminergic neuronal cells 1RB3AN_27_ (N27) were used, derived from day 12 rat fetal mesencephalic tissue and positive for tyrosine hydroxylase.^47^ N27 cells were grown in RPMI 1640 medium supplemented with 10% FBS, penicillin (100 U/mL), and streptomycin (100 μg/mL). All experiments were performed between passages 10 and 20 and at 50–80% confluence in RPMI 1640 with 1% serum.

Animals were handled in accordance with European Union legislation (European Directive 2010/63/EU). Pregnant Wistar rats (E18) were from Charles River (Denmark or UK).

### Blood–Brain Barrier Studies

Astrocytes were obtained from Biobank (HDBR) under license HTA Relevant Material, the human pericyte (HBVP) cell line was from ScienCell Research Laboratories (CA), and the human blood–brain barrier cell line (HCMEC/D3) was from Merck, Germany. Specialized cell culture medium (Dulbecco’s modified Eagle’s medium (DMEM; Corning, UK), pericyte medium (PM, ScienCell), and EndoGROTM-MV complete media kit (Millipore) were used for astrocyte, pericyte, and endothelial cell culture, respectively. Collagen I was from Sigma-Aldrich. Collagen-coated inserts (3.0 μm pore diameter, 12 mm membrane diameter) were from Corning. All types of cells were maintained at 37 ° C in a humidified incubator with 5% CO_2_ and used up to 10 passages for the experiments. Transwells contained 750 μL of medium in the basolateral compartment and 150 μL in the apical compartment.

The BBB model was used based on a modified triple-co-culture Transwell protocol developed by Stone^54^ (Figure S5a). Briefly, the upper side of 24-well Transwell inserts was coated with 150 μg/mL collagen-1 for 1 h at 37 °C. A mix of HBVP (10^4^/insert) and astrocytes (5 × 10^4^/insert) was then seeded on the basolateral side of the membrane, and cells were allowed to adhere for at least 4 h in the incubator. Thereafter, hCMEC/D3 cells (2.5 × 10^4^/insert) were seeded on the apical side of the membrane. Cells were allowed to grow in 150 μL (apical chamber) and 750 μL (basolateral chamber) of the BBB medium, composed of 50% HCMEC medium and 50% HBVP medium, for 6–7 days to reach confluence. Thereafter, the BBB integrity was assessed by measuring its permeability to dextran-rhodamine B. Briefly, the culture medium in the upper chamber was replaced with the BBB medium supplemented with 0.5 mg/mL 70 kDa dextran-rhodamine B (Life Technologies). After 4 h, the fluorescence intensity in the collector was measured using a Synergy 2 multimode microplate reader (Biotek). Samples displaying dextran-rhodamine B permeability higher than 20% of the empty control insert were discarded as previously described.^79^ The dextran concentrations in the basolateral space were determined with the measured fluorescence intensity values using a standard calibration curve, and dextran permeability coefficients were calculated (Figure S5c) using a previously described equation:^58,80^
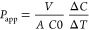
1where *V* is the volume of the sampled solution (750 μL), *A* is the surface area of the model BBB barrier (0.33 cm^2^), Δ*C*/Δ*Τ* is the concentration change in the basolateral space along time (4 h), and *C*0 is the concentration difference across the barrier (500 μg/mL).

For cell treatment, the H3-AuNP/AuNS suspended in water and ultrasonicated for 1 min wase diluted in the BBB medium to a final working concentration of 80 μg mL^–1^ and further vortexed for 5 min. The model BBB was then incubated with the H3-AuNP/NS for 48 h. Since the absorption maximum of the AuNS is highly sensitive to the spike morphology, which can vary between the batches, the spectrum of the H3-AuNS dissolved in diH_2_O was taken as a reference prior to BBB studies and used for further measurements (Figure S5b). Samples of the medium from the upper (apical) and lower (basolateral) chambers were collected and analyzed by UV–vis at wavelengths corresponding to the maximal absorbances of the H3-AuNP/AuNS batches (535 nm/730 nm, respectively). The absorbance values blanked against the BBB medium were corrected for the sample volumes, and the absorbance ratios *A*_basolateral_/*A*_apical_ × 100% were calculated for each insert and averaged for the H3-AuNP and H3-AuNS.

### Neuronal Survival and Neurite Outgrowth Assays

Rat hippocampal (E19) cultures were prepared as described in our previous articles (refs (4), (6), (81), and (84)). To evaluate neuroprotection in the oxidative stress and 6-OHDA models, hippocampal neurons (7 DIV) or N27 cells plated in poly-l-lysine coated 8-well LabTek Permanox slides (NUNC, Denmark or UK) were treated with unconjugated peptides or H3/H6-based nanoformulations, challenged with H_2_O_2_ (60 μM, hippocampal neurons) or 6-OHDA (100 μM, N27 cells, both from Sigma), further cultured for 24 h, fixed, and stained with Hoechst 33258 (1:1000; Invitrogen). Each slide also included a well with untreated cells as a control. For ROS quantification, cells were incubated with the CellROX Deep Red Reagent (5 μM; ThermoFisher Scientific, Denmark) for 30 min, fixed, and counterstained with Hoechst 33258. For the Aβ neurotoxicity assay, mature hippocampal cultures (14–15 DIV) were used and previously shown to be a reliable cell model to study AD-associated neurodegeneration.^82,83^ Dried pellets of synthetic Aβ_1-42_ (rPeptide, USA) prepared as described in our previous publications^27,28^ were dissolved in DMSO, vortexed, incubated at 37 °C for 24 h, and applied (8 μM) to cultured hippocampal neurons either alone or in combination with serially diluted peptides or nanocompounds. Neurons were further cultured for 48 h, fixed, and stained with Hoechst 33258. For fluorescence imaging, neurons were stained with polyclonal rabbit anti-growth-associated-protein-43 (GAP-43; 1:1000; Millipore, Denmark) overnight at 4 °C followed by incubation with secondary Alexa-conjugated antibodies (Invitrogen, Denmark). Cells were counterstained with Hoechst 33258 and images were acquired with an epifluorescence Olympus BX61 microscope equipped with an Olympus DP71 camera.

To evaluate neuronal survival, images of 1000–1200 cells were recorded in systematic series of fields of view across the whole area of a well for each group in each experiment employing a Nikon Eclipse E800 microscope with a Nikon Plan ×20 objective (Nikon, Tokyo, Japan) coupled to a video camera (QImaging, Surrey, Canada). For the AD model or ROS analysis, a Zeiss Axiovert 100 microscope with a Zeiss ×20 objective coupled to the AxioCam MRm camera (Zeiss) was used. Images were acquired using ImagePro (Media Cybernetics, Rockville, USA) or ZEN 2012 (Zeiss). Neuronal survival or ROS levels were evaluated as the ratio of live (non-pyknotic) or ROS-positive neurons, respectively, to the total number of cells using the PlabApp software (Protein Laboratory, University of Copenhagen, Denmark, 2002) as previously described (ref (6) and references therein). The obtained viability levels were normalized to those in untreated controls (CTL, set to 100%).

The neurite outgrowth assay was performed as in our previous studies.^4,6,84^ Freshly isolated hippocampal neurons were plated in 8-well LabTek Permanox slides (NUNC) at a density of 10,000 cells per well, stimulated with serially diluted peptides or nanocompounds, and grown for 24 h. Each slide also included a well with unstimulated cells and a well with the unconjugated H3 or H6 peptides as controls. Thereafter, hippocampal cultures were fixed with 4% formaldehyde for 30 min and stained with Coomassie Blue R250 and neurite outgrowth was evaluated using computer-assisted microscopy as described elsewhere.^4,84^

### Estimation of AuNP/NS Potency

For estimation of the H3/H6-AuNP/NS potencies in the neurite outgrowth assay (Figure 6), experimental data (neurite length *vs* NP/NS concentration) were fitted to the first-order Langmuir equation using Mathematica 8.0 software (Wolfram Research, USA):
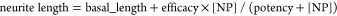
2where efficacy (the maximal neuritogenic effect of a given nanocompound) and potency (the median effective compound concentration) are the estimated parameters, basal_length is the basal length of neurites in the absence of stimulation (corresponding to [NP/NS] = 0, Figure 6), and [NP] is the nanocompound concentration ([NP/NS], Figure 6).

To estimate the H3-AuNP/NS potency in the H_2_O_2_-induced neurototoxicity assay (Figure 7B), a similar first-order Langmuir equation was used:
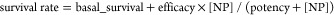
3where efficacy (the maximal prosurvival effect of a given nanocompound) and potency are the estimated parameters, basal_survival is the basal survival of the H_2_O_2_-treated neurons in the absence of nanocompounds (corresponding to [NP/NS] = 0, Figure 7B), and the rest of the parameters are defined as in eq 2.

### Evaluation of Nanocompound Toxicity In Vivo

Animal experiments were conducted in accordance with the guidelines of the Danish Animal Experimentation Inspectorate (license number: 2021-15-0201-01036) and approved by the local institutional animal care unit. Male c57/Bl6j mice (Janvier, Denmark, 24–25 g at arrival) were housed under standard climate-controlled housing conditions with a 12 h light cycle and free access to water and chow. After 1 week of acclimatization, mice were injected with probe-sonicated samples of H3/H6 spheres (i.p., 5 mg/kg) or a vehicle saline. Animals were euthanized 24 h post-injection, and blood was collected *via* cardiac puncture immediately after euthanasia. Blood plasma for biochemistry was obtained by a heparinized blood vacutainer (BD Diagnostics, Oxford, UK), centrifuged (2500*g*, 4 °C for 10 min), and analyzed using the ADVIA 1800 Chemistry System (Siemens Healthcare, Ballerup, Denmark) for standard markers of liver (ALT, AST, ALP) and kidney (BUN) damage.

### Statistics

Statistic analysis was performed using Origin 8 software (OriginLab) and GraphPad Prism 6 (GraphPad software, Inc., La Jolla, CA, USA) by two-tailed *t* test, one-way ANOVA or two-way ANOVA with Tukey’s, and Dunnett or Sidak post-tests to identify statistically significant groups. Unless indicated otherwise, results are expressed as means ± SEM, *p < 0.05; **p < 0.01; ***p < 0.001.

## Supporting Information Available

Figure S1: UV-vis absorbance of non-conjugated and peptide-functionalized nanospheres and nanostars; Figure S2: UV-vis absorbance calibration graphs for the H3 and H6 peptides, the time course of the peptide release from the H3- or H6-functionalized nanospheres, and DLS size distribution analysis of the naked and H3-conjugated nanospheres; Figure S3: XPS reference datasets of PEG, H3, and H6; Figure S4: XPS survey spectra of AuNP and AuNS samples; Figure S5: schematic representation and characteristics of the BBB model (PDF)

## ABBREVIATIONS

6-OHDA: 6*-*hydroxydopamine
AD: Alzheimer’s disease
AuNP/NS: gold nanoparticles/nanostars
BBB: blood–brain barrier
CNS: central nervous system
PD: Parkinson’s disease
PEG: polyethyleneglycol

## References

[ref1] Duro-Castano A.; Moreira Leite D.; Forth J.; Deng Y.; Matias D.; Noble Jesus C.; Battaglia G. Designing peptide nanoparticles for efficient brain delivery. Adv. Drug Delivery Rev. 2020, 160, 52–77. 10.1016/j.addr.2020.10.001. 33031897

[ref2] Theodorou I. G.; Jiang Q.; Malms L.; Xie X.; Coombes R. C.; Aboagye E. O.; Porter A. E.; Ryan M. P.; Xie F. Fluorescence enhancement from single gold nanostars: towards ultra-bright emission in the first and second near-infrared biological windows. Nanoscale 2018, 10, 15854–15864. 10.1039/c8nr04567d. 30105338

[ref3] Theodorou I. G.; Ruenraroengsak P.; Gonzalez-Carter D. A.; Jiang Q.; Yagüe E.; Aboagye E. O.; Coombes R. C.; Porter A. E.; Ryan M. P.; Xie F. Towards multiplexed near-infrared cellular imaging using gold nanostar arrays with tunable fluorescence enhancement. Nanoscale 2019, 11, 2079–2088. 10.1039/c8nr09409h. 30648720

[ref4] Dmytriyeva O.; Pankratova S.; Owczarek S.; Sonn K.; Soroka V.; Ridley C. M.; Marsolais A.; Lopez-Hoyos M.; Ambartsumian N.; Lukanidin E.; Bock E.; Berezin V.; Kiryushko D. The metastasis-promoting S100A4 protein confers neuroprotection in brain injury. Nat. Commun. 2012, 3, 1197 10.1038/ncomms2202. 23149742

[ref5] Moldovan M.; Pinchenko V.; Dmytriyeva O.; Pankratova S.; Fugleholm K.; Klingelhofer J.; Bock E.; Berezin V.; Krarup C.; Kiryushko D. Peptide mimetic of the S100A4 protein modulates peripheral nerve regeneration and attenuates the progression of neuropathy in myelin protein P0 null mice. Mol. Med. 2013, 19, 43–53. 10.2119/molmed.2012.00248. 23508572 PMC3646097

[ref6] Pankratova S.; Klingelhofer J.; Dmytriyeva O.; Owczarek S.; Renziehausen A.; Syed N.; Porter A. E.; Dexter D. T.; Kiryushko D. The S100A4 Protein Signals through the ErbB4 Receptor to Promote Neuronal Survival. Theranostics 2018, 8, 3977–3990. 10.7150/thno.22274. 30083275 PMC6071530

[ref7] Gonzalez-Carter D. A.; Ong Z. Y.; McGilvery C. M.; Dunlop I. E.; Dexter D. T.; Porter A. E. L-DOPA functionalized, multi-branched gold nanoparticles as brain-targeted nano-vehicles. Nanomedicine 2019, 15, 1–11. 10.1016/j.nano.2018.08.011. 30189294

[ref8] Valente P.; Kiryushko D.; Sacchetti S.; Machado P.; Cobley C. M.; Mangini V.; Porter A. E.; Spatz J. P.; Fleck R. A.; Benfenati F.; Fiammengo R. Conopeptide-Functionalized Nanoparticles Selectively Antagonize Extrasynaptic N-Methyl-d-aspartate Receptors and Protect Hippocampal Neurons from Excitotoxicity In Vitro. ACS Nano 2020, 14, 6866–6877. 10.1021/acsnano.0c00866. 32510204

[ref9] Aberg F.; Kozlova E. N. Metastasis-associated mts1 (S100A4) protein in the developing and adult central nervous system. J. Comp. Neurol. 2000, 424, 269–282. 10.1002/1096-9861(20000821)424:2<269::AID-CNE6>3.0.CO;2-M. 10906702

[ref10] Kobori N.; Clifton G. L.; Dash P. Altered expression of novel genes in the cerebral cortex following experimental brain injury. Brain Res. Mol. Brain Res. 2002, 104, 148–158. 10.1016/S0169-328X(02)00331-5. 12225869

[ref11] Kozlova E. N.; Lukanidin E. Mts1 protein expression in the central nervous system after injury. Glia 2002, 37, 337–348. 10.1002/glia.10045. 11870873

[ref12] Mei L.; Xiong W. C. Neuregulin 1 in neural development, synaptic plasticity and schizophrenia. Nat. Rev. Neurosci. 2008, 9, 437–452. 10.1038/nrn2392. 18478032 PMC2682371

[ref13] Ryu J.; Yu H. N.; Cho H.; Kim H. S.; Baik T. K.; Lee S. J.; Woo R. S. Neuregulin-1 exerts protective effects against neurotoxicities induced by C-terminal fragments of APP via ErbB4 receptor. J. Pharmacol. Sci. 2012, 119, 73–81. 10.1254/jphs.12057FP. 22739235

[ref14] Ghashghaei H. T.; Weber J.; Pevny L.; Schmid R.; Schwab M. H.; Kent Lloyd K. C.; Eisenstat D. D.; Lai C.; Anton E. S. The role of neuregulin-ErbB4 interactions on the proliferation and organization of cells in the subventricular zone. Proc. Natl. Acad. Sci. U. S. A. 2006, 103, 1930–1935. 10.1073/pnas.0510410103. 16446434 PMC1413654

[ref15] Zhang L.; Fletcher-Turner A.; Marchionni M. A.; Apparsundaram S.; Lundgren K. H.; Yurek D. M.; Seroogy K. B. Neurotrophic and neuroprotective effects of the neuregulin glial growth factor-2 on dopaminergic neurons in rat primary midbrain cultures. J. Neurochem. 2004, 91, 1358–1368. 10.1111/j.1471-4159.2004.02817.x. 15584912

[ref16] Gerecke K. M.; Wyss J. M.; Carroll S. L. Neuregulin-1beta induces neurite extension and arborization in cultured hippocampal neurons. Mol. Cell. Neurosci. 2004, 27, 379–393. 10.1016/j.mcn.2004.08.001. 15555917

[ref17] Min S. S.; An J.; Lee J. H.; Seol G. H.; Im J. H.; Kim H. S.; Baik T. K.; Woo R. S. Neuregulin-1 prevents amyloid beta-induced impairment of long-term potentiation in hippocampal slices via ErbB4. Neurosci. Lett. 2011, 505, 6–9. 10.1016/j.neulet.2011.05.246. 21787838

[ref18] Carlsson T.; Schindler F. R.; Hollerhage M.; Depboylu C.; Arias-Carrion O.; Schnurrbusch S.; Rosler T. W.; Wozny W.; Schwall G. P.; Groebe K.; Oertel W. H.; Brundin P.; Schrattenholz A.; Hoglinger G. U. Systemic administration of neuregulin-1beta1 protects dopaminergic neurons in a mouse model of Parkinson’s disease. J. Neurochem. 2011, 117, 1066–1074. 10.1111/j.1471-4159.2011.07284.x. 21517849

[ref19] Iwakura Y.; Nawa H. ErbB1-4-dependent EGF/neuregulin signals and their cross talk in the central nervous system: pathological implications in schizophrenia and Parkinson’s disease. Front. Cell. Neurosci. 2013, 7, 4 10.3389/fncel.2013.00004. 23408472 PMC3570895

[ref20] Guo W. P.; Wang J.; Li R. X.; Peng Y. W. Neuroprotective effects of neuregulin-1 in rat models of focal cerebral ischemia. Brain Res. 2006, 1087, 180–185. 10.1016/j.brainres.2006.03.007. 16616052

[ref21] Depboylu C.; Rosler T. W.; de Andrade A.; Oertel W. H.; Hoglinger G. U. Systemically administered neuregulin-1beta1 rescues nigral dopaminergic neurons via the ErbB4 receptor tyrosine kinase in MPTP mouse models of Parkinson’s disease. J. Neurochem. 2015, 133, 590–597. 10.1111/jnc.13026. 25581060

[ref22] Kiryushko D.; Novitskaya V.; Soroka V.; Klingelhofer J.; Lukanidin E.; Berezin V.; Bock E. Molecular mechanisms of Ca(2+) signaling in neurons induced by the S100A4 protein. Mol. Cell. Biol. 2006, 26, 3625–3638. 10.1128/MCB.26.9.3625-3638.2006. 16612001 PMC1447425

[ref23] Novitskaya V.; Grigorian M.; Kriajevska M.; Tarabykina S.; Bronstein I.; Berezin V.; Bock E.; Lukanidin E. Oligomeric forms of the metastasis-related Mts1 (S100A4) protein stimulate neuronal differentiation in cultures of rat hippocampal neurons. J. Biol. Chem/ 2000, 275, 41278–41286. 10.1074/jbc.M007058200. 11018041

[ref24] Cui W.; Tao J.; Wang Z.; Ren M.; Zhang Y.; Sun Y.; Peng Y.; Li R. Neuregulin1beta1 antagonizes apoptosis via ErbB4-dependent activation of PI3-kinase/Akt in APP/PS1 transgenic mice. Neurochem. Res. 2013, 38, 2237–2246. 10.1007/s11064-013-1131-z. 23982319

[ref25] Woo R. S.; Lee J. H.; Yu H. N.; Song D. Y.; Baik T. K. Expression of ErbB4 in the neurons of Alzheimer’s disease brain and APP/PS1 mice, a model of Alzheimer’s disease. Anat. Cell. Biol. 2011, 44, 116–127. 10.5115/acb.2011.44.2.116. 21829755 PMC3145840

[ref26] Zhang H.; Zhang L.; Zhou D.; He X.; Wang D.; Pan H.; Zhang X.; Mei Y.; Qian Q.; Zheng T.; Jones F. E.; Sun B. Ablating ErbB4 in PV neurons attenuates synaptic and cognitive deficits in an animal model of Alzheimer’s disease. Neurobiol. Dis. 2017, 106, 171–180. 10.1016/j.nbd.2017.07.001. 28684271

[ref27] Kubánková M.; López-Duarte I.; Kiryushko D.; Kuimova M. K. Molecular rotors report on changes in live cell plasma membrane microviscosity upon interaction with beta-amyloid aggregates. Soft Matter 2018, 14, 9466–9474. 10.1039/c8sm01633j. 30427370

[ref28] Kubánková M.; Summers P. A.; López-Duarte I.; Kiryushko D.; Kuimova M. K. Microscopic Viscosity of Neuronal Plasma Membranes Measured Using Fluorescent Molecular Rotors: Effects of Oxidative Stress and Neuroprotection. ACS Appl. Mater. Interfaces 2019, 11, 36307–36315. 10.1021/acsami.9b10426. 31513373

[ref29] Wiley D. T.; Webster P.; Gale A.; Davis M. E. Transcytosis and brain uptake of transferrin-containing nanoparticles by tuning avidity to transferrin receptor. Proc. Natl. Acad. Sci. U. S. A. 2013, 110, 8662–8667. 10.1073/pnas.1307152110. 23650374 PMC3666717

[ref30] Lopes Rodrigues R.; Xie F.; Porter A. E.; Ryan M. P. Geometry-induced protein reorientation on the spikes of plasmonic gold nanostars. Nanoscale Adv. 2020, 2, 1144–1151. 10.1039/C9NA00584F. 36133070 PMC9418033

[ref31] Haiss W.; Thanh N. T. K.; Aveyard J.; Fernig D. G. Determination of size and concentration of gold nanoparticles from UV-vis spectra. Anal. Chem. 2007, 79, 4215–4221. 10.1021/ac0702084. 17458937

[ref32] Makhsin S. R.; Razak K. A.; Noordin R.; Zakaria N. D.; Chun T. S. The effects of size and synthesis methods of gold nanoparticle-conjugated MαHIgG4 for use in an immunochromatographic strip test to detect brugian filariasis. Nanotechnology 2012, 23, 495719 10.1088/0957-4484/23/49/495719. 23164811

[ref33] Turecek P. L.; Bossard M. J.; Schoetens F.; Ivens I. A. PEGylation of Biopharmaceuticals: A Review of Chemistry and Nonclinical Safety Information of Approved Drugs. J. Pharm. Sci. 2016, 105, 460–475. 10.1016/j.xphs.2015.11.015. 26869412

[ref34] Lee E.; Jeon H.; Lee M.; Ryu J.; Kang C.; Kim S.; Jung J.; Kwon Y. Molecular origin of AuNPs-induced cytotoxicity and mechanistic study. Sci. Rep. 2019, 9, 2494–2494. 10.1038/s41598-019-39579-3. 30792478 PMC6385177

[ref35] Pi J. M.; Stella M.; Fernando N. K.; Lam A. Y.; Regoutz A.; Ratcliff L. E. Predicting Core Level Photoelectron Spectra of Amino Acids Using Density Functional Theory. J. Phys. Chem. Lett. 2020, 11, 2256–2262. 10.1021/acs.jpclett.0c00333. 32125160

[ref36] Regoutz A.; Wolinska M. S.; Fernando N. K.; Ratcliff L. E. A combined density functional theory and x-ray photoelectron spectroscopy study of the aromatic amino acids. Electron. Struct. 2020, 2, 044005 10.1088/2516-1075/abd63c.

[ref37] Boye K.; Maelandsmo G. M. S100A4 and metastasis: a small actor playing many roles. Am. J. Pathol. 2010, 176, 528–535. 10.2353/ajpath.2010.090526. 20019188 PMC2808059

[ref38] Bramlett H. M.; Dietrich W. D. Pathophysiology of cerebral ischemia and brain trauma: similarities and differences. J. Cereb. Blood Flow Metab. 2004, 24, 133–150. 10.1097/01.WCB.0000111614.19196.04. 14747740

[ref39] Cheng Y.; Loh Y. P.; Birch N. P. Neuroserpin Attenuates H2O2-Induced Oxidative Stress in Hippocampal Neurons via AKT and BCL-2 Signaling Pathways. J. Mol. Neurosci. 2017, 61, 123–131. 10.1007/s12031-016-0807-7. 27510267

[ref40] Sies H. Hydrogen peroxide as a central redox signaling molecule in physiological oxidative stress: Oxidative eustress. Redox. Biol. 2017, 11, 613–619. 10.1016/j.redox.2016.12.035. 28110218 PMC5256672

[ref41] Jo H. S.; Kim D. W.; Shin M. J.; Cho S. B.; Park J. H.; Lee C. H.; Yeo E. J.; Choi Y. J.; Yeo H. J.; Sohn E. J.; Son O.; Cho S. W.; Kim D. S.; Yu Y. H.; Lee K. W.; Park J.; Eum W. S.; Choi S. Y. Tat-HSP22 inhibits oxidative stress-induced hippocampal neuronal cell death by regulation of the mitochondrial pathway. Mol. Brain 2017, 10, 1 10.1186/s13041-016-0281-8. 28052764 PMC5210279

[ref42] Pedersen M. V.; Køhler L. B.; Grigorian M.; Novitskaya V.; Bock E.; Lukanidin E.; Berezin V. The Mts1/S100A4 protein is a neuroprotectant. J. Neurosci. Res. 2004, 77, 777–786. 10.1002/jnr.20221. 15334597

[ref43] Blesa J.; Phani S.; Jackson-Lewis V.; Przedborski S. Classic and new animal models of Parkinson’s disease. J. Biomed. Biotechnol. 2012, 2012, 845618 10.1155/2012/845618. 22536024 PMC3321500

[ref44] Xu Q.; Kanthasamy A. G.; Jin H.; Reddy M. B. Hepcidin Plays a Key Role in 6-OHDA Induced Iron Overload and Apoptotic Cell Death in a Cell Culture Model of Parkinson’s Disease. Parkinson’s Dis. 2016, 2016, 8684130 10.1155/2016/8684130. 27298749 PMC4889865

[ref45] Dranka B. P.; Zielonka J.; Kanthasamy A. G.; Kalyanaraman B. Alterations in bioenergetic function induced by Parkinson’s disease mimetic compounds: lack of correlation with superoxide generation. J. Neurochem. 2012, 122, 941–951. 10.1111/j.1471-4159.2012.07836.x. 22708893 PMC3423581

[ref46] Offenburger S. L.; Jongsma E.; Gartner A. Mutations in Caenorhabditis elegans neuroligin-like glit-1, the apoptosis pathway and the calcium chaperone crt-1 increase dopaminergic neurodegeneration after 6-OHDA treatment. PLoS Genet. 2018, 14, e1007106 10.1371/journal.pgen.1007106. 29346364 PMC5773152

[ref47] Adams F. S.; La Rosa F. G.; Kumar S.; Edwards-Prasad J.; Kentroti S.; Vernadakis A.; Freed C. R.; Prasad K. N. Characterization and transplantation of two neuronal cell lines with dopaminergic properties. Neurochem. Res. 1996, 21, 619–627. 10.1007/BF02527762. 8726972

[ref48] Prasad K. N.; Clarkson E. D.; La Rosa F. G.; Edwards-Prasad J.; Freed C. R. Efficacy of grafted immortalized dopamine neurons in an animal model of parkinsonism: a review. Mol. Genet. Metab. 1998, 65, 1–9. 10.1006/mgme.1998.2726. 9787089

[ref49] Anantharam V.; Kitazawa M.; Wagner J.; Kaul S.; Kanthasamy A. G. Caspase-3-dependent proteolytic cleavage of protein kinase Cdelta is essential for oxidative stress-mediated dopaminergic cell death after exposure to methylcyclopentadienyl manganese tricarbonyl. J. Neurosci. 2002, 22, 1738–1751. 10.1523/JNEUROSCI.22-05-01738.2002. 11880503 PMC6758879

[ref50] Cantu D.; Fulton R. E.; Drechsel D. A.; Patel M. Mitochondrial aconitase knockdown attenuates paraquat-induced dopaminergic cell death via decreased cellular metabolism and release of iron and H(2)O(2). J. Neurochem. 2011, 118, 79–92. 10.1111/j.1471-4159.2011.07290.x. 21517855 PMC3182850

[ref51] Choi D. H.; Kim Y. J.; Kim Y. G.; Joh T. H.; Beal M. F.; Kim Y. S. Role of matrix metalloproteinase 3-mediated alpha-synuclein cleavage in dopaminergic cell death. J. Biol. Chem. 2011, 286, 14168–14177. 10.1074/jbc.M111.222430. 21330369 PMC3077618

[ref52] Dias V.; Junn E.; Mouradian M. M. The role of oxidative stress in Parkinson’s disease. J. Parkinson’s Dis. 2013, 3, 461–491. 10.3233/JPD-130230. 24252804 PMC4135313

[ref53] Schieber M.; Chandel N. S. ROS function in redox signaling and oxidative stress. Curr. Biol. 2014, 24, R453–R462. 10.1016/j.cub.2014.03.034. 24845678 PMC4055301

[ref54] Stone N. L.; England T. J.; O’Sullivan S. E. A Novel Transwell Blood Brain Barrier Model Using Primary Human Cells. Front. Cell. Neurosci. 2019, 13, 230 10.3389/fncel.2019.00230. 31244605 PMC6563620

[ref55] Stalinska J.; Vittori C.; Ingraham Iv C. H.; Carson S. C.; Plaisance-Bonstaff K.; Lassak A.; Faia C.; Colley S. B.; Peruzzi F.; Reiss K.; Jursic B. S. Anti-glioblastoma effects of phenolic variants of benzoylphenoxyacetamide (BPA) with high potential for blood brain barrier penetration. Sci. Rep. 2022, 12, 3384 10.1038/s41598-022-07247-8. 35232976 PMC8888627

[ref56] Kadir R. R. A.; Alwjwaj M.; McCarthy Z.; Bayraktutan U. Therapeutic hypothermia augments the restorative effects of PKC-β and Nox2 inhibition on an in vitro model of human blood-brain barrier. Metab. Brain Dis. 2021, 36, 1817–1832. 10.1007/s11011-021-00810-8. 34398388 PMC8437893

[ref57] Nevo N.; Chossat N.; Gosgnach W.; Logeart D.; Mercadier J. J.; Michel J. B. Increasing endothelial cell permeability improves the efficiency of myocyte adenoviral vector infection. J. Gene Med. 2001, 3, 42–50. 10.1002/1521-2254(2000)9999:9999<::aid-jgm149>3.0.co;2-a. 11269335

[ref58] Gray K. M.; Jung J. W.; Inglut C. T.; Huang H. C.; Stroka K. M. Quantitatively relating brain endothelial cell-cell junction phenotype to global and local barrier properties under varied culture conditions via the Junction Analyzer Program. Fluids Barriers CNS 2020, 17, 16 10.1186/s12987-020-0177-y. 32046757 PMC7014765

[ref59] Salinas K.; Kereselidze Z.; DeLuna F.; Peralta X. G.; Santamaria F. Transient extracellular application of gold nanostars increases hippocampal neuronal activity. J. Nanobiotechnol. 2014, 12, 31–31. 10.1186/s12951-014-0031-y. PMC4422288 25135485

[ref60] Chaudhury A. R.; Gerecke K. M.; Wyss J. M.; Morgan D. G.; Gordon M. N.; Carroll S. L. Neuregulin-1 and erbB4 immunoreactivity is associated with neuritic plaques in Alzheimer disease brain and in a transgenic model of Alzheimer disease. J. Neuropathol. Exp. Neurol. 2003, 62, 42–54. 10.1093/jnen/62.1.42. 12528817

[ref61] Cristóvão J. S.; Gomes C. M. S100 Proteins in Alzheimer’s Disease. Front. Neurosci. 2019, 13, 463 10.3389/fnins.2019.00463. 31156365 PMC6532343

[ref62] Li C.; Sun T.; Jiang C. Recent advances in nanomedicines for the treatment of ischemic stroke. Acta Pharm. Sin. B 2021, 11, 1767–1788. 10.1016/j.apsb.2020.11.019. 34386320 PMC8343119

[ref63] Zhang D.; Liu L.; Wang J.; Zhang H.; Zhang Z.; Xing G.; Wang X.; Liu M. Drug-loaded PEG-PLGA nanoparticles for cancer treatment. Front. Pharmacol. 2022, 13, 990505 10.3389/fphar.2022.990505. 36059964 PMC9437283

[ref64] Cui Y.; Li X.; Zeljic K.; Shan S.; Qiu Z.; Wang Z. Effect of PEGylated Magnetic PLGA-PEI Nanoparticles on Primary Hippocampal Neurons: Reduced Nanoneurotoxicity and Enhanced Transfection Efficiency with Magnetofection. ACS Appl. Mater. Interfaces 2019, 11, 38190–38204. 10.1021/acsami.9b15014. 31550131

[ref65] Zhou M.; Zhang T.; Zhang B.; Zhang X.; Gao S.; Zhang T.; Li S.; Cai X.; Lin Y. A DNA Nanostructure-Based Neuroprotectant against Neuronal Apoptosis via Inhibiting Toll-like Receptor 2 Signaling Pathway in Acute Ischemic Stroke. ACS Nano 2022, 16, 1456–1470. 10.1021/acsnano.1c09626. 34967217

[ref66] Qin X.; Xiao L.; Li N.; Hou C.; Li W.; Li J.; Yan N.; Lin Y. Tetrahedral framework nucleic acids-based delivery of microRNA-155 inhibits choroidal neovascularization by regulating the polarization of macrophages. Bioactive Mater. 2022, 14, 134–144. 10.1016/j.bioactmat.2021.11.031. PMC8892086 35310341

[ref67] Hersh A. M.; Alomari S.; Tyler B. M. Crossing the Blood-Brain Barrier: Advances in Nanoparticle Technology for Drug Delivery in Neuro-Oncology. Int. J. Mol. Sci. 2022**,** 23 (), 10.3390/ijms23084153. 10.3390/ijms23084153 PMC9032478 35456971

[ref68] Male D.; Gromnicova R.; McQuaid C. Gold Nanoparticles for Imaging and Drug Transport to the CNS. Int. Rev. Neurobiol. 2016, 130, 155–198. 10.1016/bs.irn.2016.05.003. 27678177

[ref69] Ohta S.; Kikuchi E.; Ishijima A.; Azuma T.; Sakuma I.; Ito T. Investigating the optimum size of nanoparticles for their delivery into the brain assisted by focused ultrasound-induced blood–brain barrier opening. Sci. Rep. 2020, 10, 18220 10.1038/s41598-020-75253-9. 33106562 PMC7588485

[ref70] Guerrero S.; Araya E.; Fiedler J. L.; Arias J. I.; Adura C.; Albericio F.; Giralt E.; Arias J. L.; Fernández M. S.; Kogan M. J. Improving the brain delivery of gold nanoparticles by conjugation with an amphipathic peptide. Nanomedicine 2010, 5, 897–913. 10.2217/nnm.10.74. 20735225

[ref71] Wu L. P.; Ahmadvand D.; Su J.; Hall A.; Tan X.; Farhangrazi Z. S.; Moghimi S. M. Crossing the blood-brain-barrier with nanoligand drug carriers self-assembled from a phage display peptide. Nat. Commun. 2019, 10, 4635 10.1038/s41467-019-12554-2. 31604928 PMC6789111

[ref72] Han L.; Jiang C. Evolution of blood–brain barrier in brain diseases and related systemic nanoscale brain-targeting drug delivery strategies. Acta Pharmaceutica Sinica B 2021, 11, 2306–2325. 10.1016/j.apsb.2020.11.023. 34522589 PMC8424230

[ref73] Yin T.; Xie W.; Sun J.; Yang L.; Liu J. Penetratin Peptide-Functionalized Gold Nanostars: Enhanced BBB Permeability and NIR Photothermal Treatment of Alzheimer’s Disease Using Ultralow Irradiance. ACS Appl. Mater. Interfaces 2016, 8, 19291–19302. 10.1021/acsami.6b05089. 27411476

[ref74] Popovtzer R.; Agrawal A.; Kotov N. A.; Popovtzer A.; Balter J.; Carey T. E.; Kopelman R. Targeted gold nanoparticles enable molecular CT imaging of cancer. Nano Lett. 2008, 8, 4593–4596. 10.1021/nl8029114. 19367807 PMC2772154

[ref75] Yuan H.; Khoury C. G.; Hwang H.; Wilson C. M.; Grant G. A.; Vo-Dinh T. Gold nanostars: surfactant-free synthesis, 3D modelling, and two-photon photoluminescence imaging. Nanotechnology 2012, 23, 075102 10.1088/0957-4484/23/7/075102. 22260928 PMC3400343

[ref76] Cruz-Matías I.; Ayala D.; Hiller D.; Gutsch S.; Zacharias M.; Estradé S.; Peiró F. Sphericity and roundness computation for particles using the extreme vertices model. J. Comput. Sci. 2019, 30, 28–40. 10.1016/j.jocs.2018.11.005.

[ref77] Kremer J. R.; Mastronarde D. N.; McIntosh J. R. Computer visualization of three-dimensional image data using IMOD. J. Struct. Biol. 1996, 116, 71–76. 10.1006/jsbi.1996.0013. 8742726

[ref78] Mastronarde D. N.; Held S. R. Automated tilt series alignment and tomographic reconstruction in IMOD. J. Struct. Biol. 2017, 197, 102–113. 10.1016/j.jsb.2016.07.011. 27444392 PMC5247408

[ref79] Proust A.; Barat C.; Leboeuf M.; Drouin J.; Tremblay M. J. Contrasting effect of the latency-reversing agents bryostatin-1 and JQ1 on astrocyte-mediated neuroinflammation and brain neutrophil invasion. J. Neuroinflammation 2017, 14, 242 10.1186/s12974-017-1019-y. 29228979 PMC5725742

[ref80] Ahn S. I.; Sei Y. J.; Park H.-J.; Kim J.; Ryu Y.; Choi J. J.; Sung H.-J.; MacDonald T. J.; Levey A. I.; Kim Y. Microengineered human blood–brain barrier platform for understanding nanoparticle transport mechanisms. Nat. Commun. 2020, 11, 175 10.1038/s41467-019-13896-7. 31924752 PMC6954233

[ref81] Soroka V.; Kiryushko D.; Novitskaya V.; Ronn L. C.; Poulsen F. M.; Holm A.; Bock E.; Berezin V. Induction of neuronal differentiation by a peptide corresponding to the homophilic binding site of the second Ig module of the neural cell adhesion molecule. J. Biol. Chem. 2002, 277, 24676–24683. 10.1074/jbc.M109694200. 11983682

[ref82] Jin M.; Shepardson N.; Yang T.; Chen G.; Walsh D.; Selkoe D. J. Soluble amyloid beta-protein dimers isolated from Alzheimer cortex directly induce Tau hyperphosphorylation and neuritic degeneration. Proc. Natl. Acad. Sci. U. S. A. 2011, 108, 5819–5824. 10.1073/pnas.1017033108. 21421841 PMC3078381

[ref83] Chang L.; Cui W.; Yang Y.; Xu S.; Zhou W.; Fu H.; Hu S.; Mak S.; Hu J.; Wang Q.; Pui-Yan Ma V.; Choi T. C.; Ma E. D.; Tao L.; Pang Y.; Rowan M. J.; Anwyl R.; Han Y.; Wang Q. Protection against β-amyloid-induced synaptic and memory impairments via altering β-amyloid assembly by bis(heptyl)-cognitin. Sci. Rep. 2015, 5, 10256 10.1038/srep10256. 26194093 PMC4508546

[ref84] Pankratova S.; Kiryushko D.; Sonn K.; Soroka V.; Kohler L. B.; Rathje M.; Gu B.; Gotfryd K.; Clausen O.; Zharkovsky A.; Bock E.; Berezin V. Neuroprotective properties of a novel, non-haematopoietic agonist of the erythropoietin receptor. Brain 2010, 133, 2281–2294. 10.1093/brain/awq101. 20435631

