# Supplementary material for: Nanostars Carrying Multifunctional Neurotrophic Dendrimers Protect Neurons in Preclinical In Vitro Models of Neurodegenerative Disorders
Source: ACS Appl Mater Interfaces. 2022 Oct 11;14(42):47445–60. doi: 10.1021/acsami.2c14220 (PMC9614720; doi:10.1021/acsami.2c14220)
Supplement: Supplementary file 1 — am2c14220_si_001.pdf [file am2c14220_si_001.pdf]

Supporting Information

Nanostars carrying multifunctional neurotrophic dendrimers protect neurons in pre-clinical *in vitro* models of neurodegenerative disorders

Corinne Morfill[1], Stanislava Pankratova[2,3], Pedro Machado[4], Nathalie K. Fernando[5], Anna Regoutz[5], Federica Talamona[1], Alessandra Pinna[1,6], Michal Klosowski[1], Robert J. Wilkinson[6,7], Roland A. Fleck[4], Fang Xie[1], Alexandra E. Porter[1*], and Darya Kiryushko[1,8,9*]

[1]*Department of Materials and London Centre for Nanotechnology, Imperial College, Exhibition Road, SW72AZ London, UK*
[2] *Department of Neuroscience, Faculty of Health and Medical Sciences, University of Copenhagen, Copenhagen 2200N, Denmark*
[3] *Comparative Paediatrics and Nutrition, Department of Veterinary and Animal Sciences, Faculty of Health and Medical Sciences, University of Copenhagen, Copenhagen, Denmark*
[4] *Centre for Ultrastructural Imaging, Kings College London, London SE1 1UL, United Kingdom*
[5] *Department of Chemistry, University College London, 20 Gordon Street, WC1H 0AJ London, UK*
[6] *The Francis Crick Institute, NW11 AT, London, UK7*[9] *Imperial College London SW7 2AZ,UK*
[7] *Imperial College London SW7 2AZ, UK*
[8]*Centre for Neuroinflammation and Neurodegeneration, Imperial College London, Hammersmith Hospital Campus, Burlington Danes Building, 160 Du Cane Road, W12 0NN London, UK*
[9]*Experimental Solid State Physics Group, Department of Physics, Imperial College, Exhibition Road, SW72AZ London, UK*

*Corresponding author: d.kiryushko@imperial.ac.uk

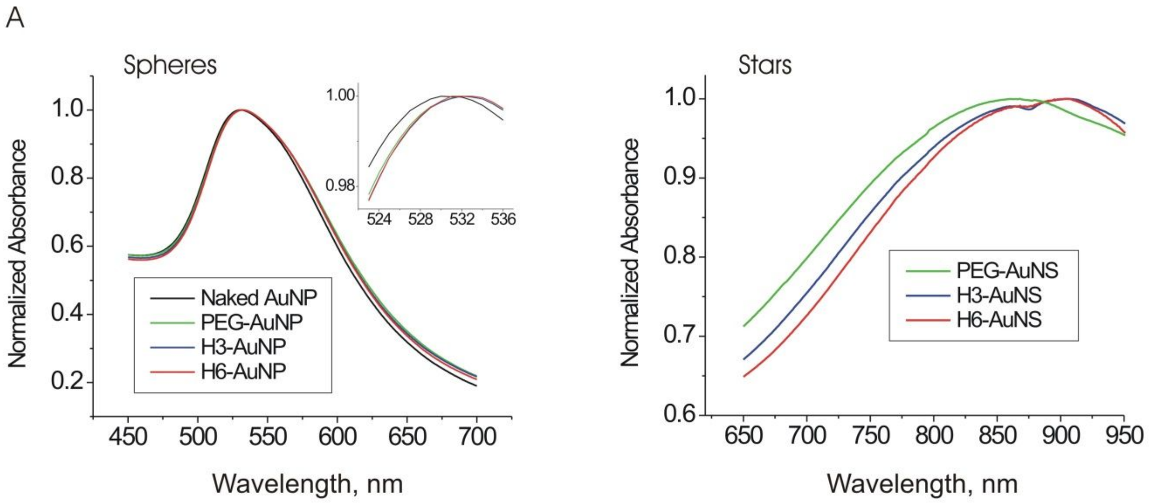

Figure S1: UV-vis absorbance of non-conjugated and peptide-functionalized nanospheres and nanostars. Left, inset, the peak absorbances of the AuNP compounds, enlarged from the main graph.

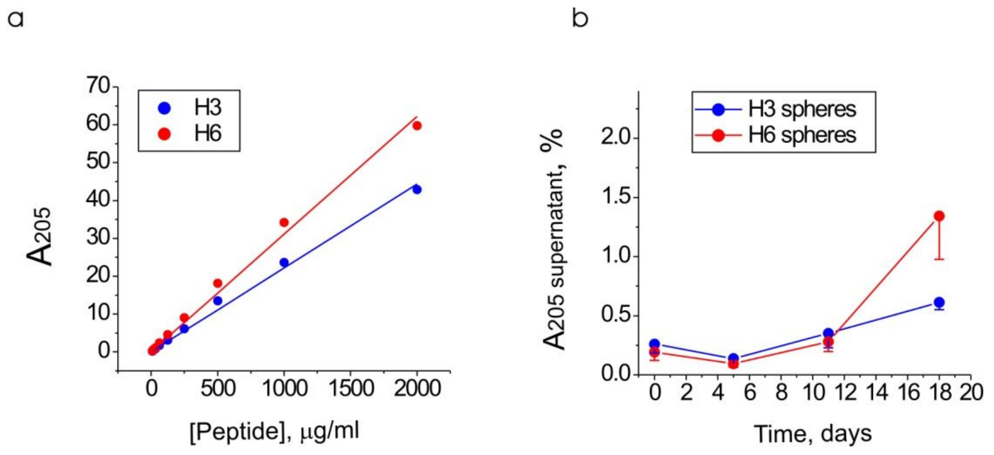

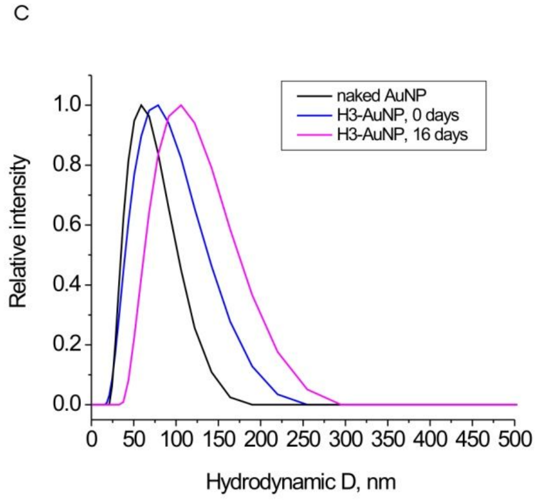

Nanostructure	Z-average, nm
AuNP naked	53.5 +/- 0.4
H3-AuNP, 0 d	76.4 +/- 0.4
H3-AuNP, 16 d	101.6 +/- 1.7

Figure S2: (a) UV-vis absorbance calibration graphs for the H3 and H6 peptides. Peptide absorbance at 205 nm (A_{205}, Y-axis) shows a linear dependence on the peptide concentration. (b) The amount of the peptide released from the H3- or H6-functionalized nanospheres as a percentage of the initial peptide concentration in the nanoparticle suspension. Time course studies, 2 independent experiments. (c) Dynamic light scattering (DLS) size distribution analysis by intensity of the naked and H3-conjugated nanospheres in deionized water immediately after (day 0) and 16 days after preparation. Representative of 2 independent experiments. Z-average was determined from 3 measurements.

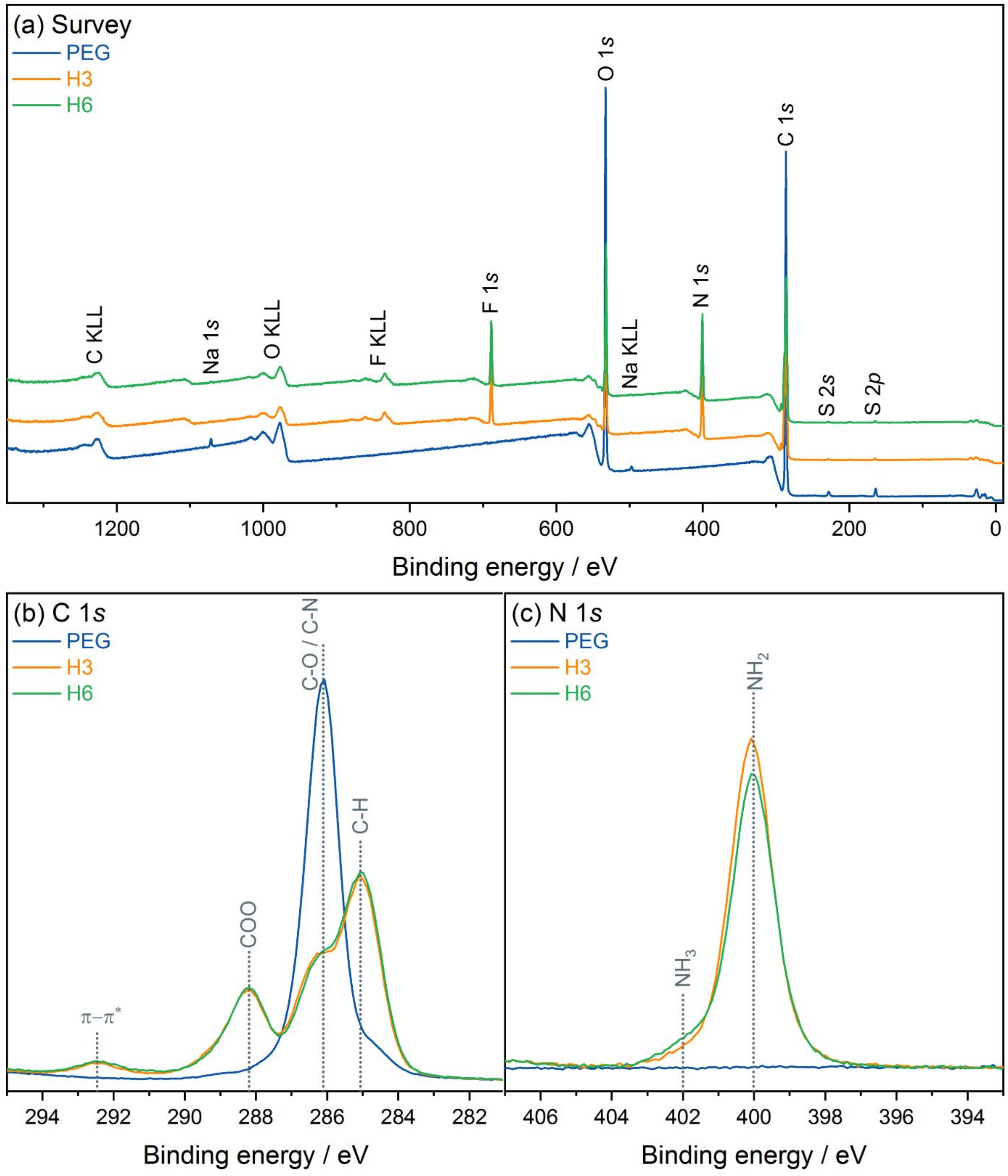

Figure S3: X-ray photoelectron spectroscopy (XPS) reference datasets of PEG, H3 and H6, including (a) Survey, (b) C 1*s* core level, and (c) N 1*s* core level spectra. In the survey spectra all major signals are indicated and for the core levels chemical states are labelled.

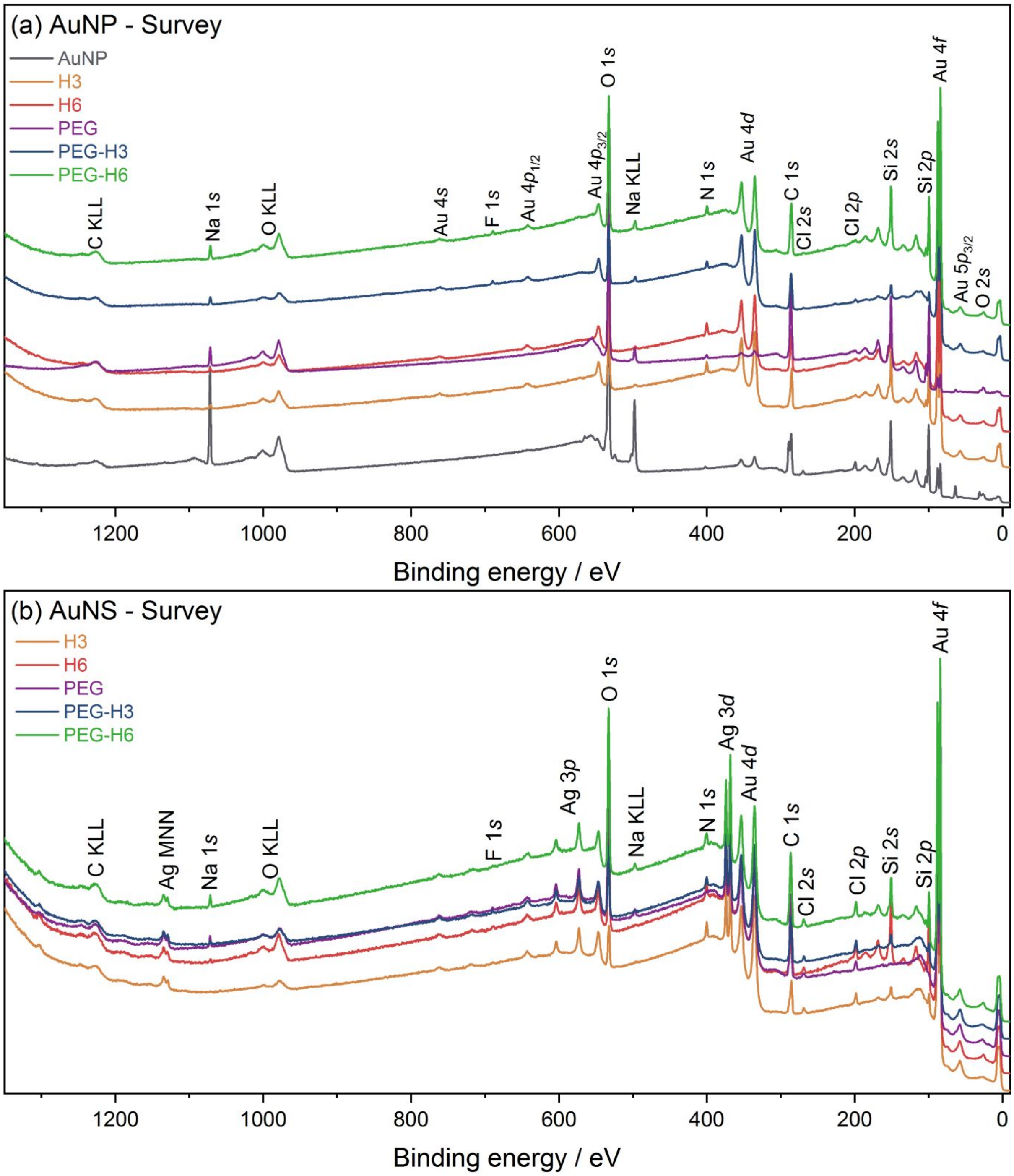

Figure S4: XPS survey spectra of (a) AuNP and (b) AuNS samples. All major signals are indicated including core states and Auger lines.

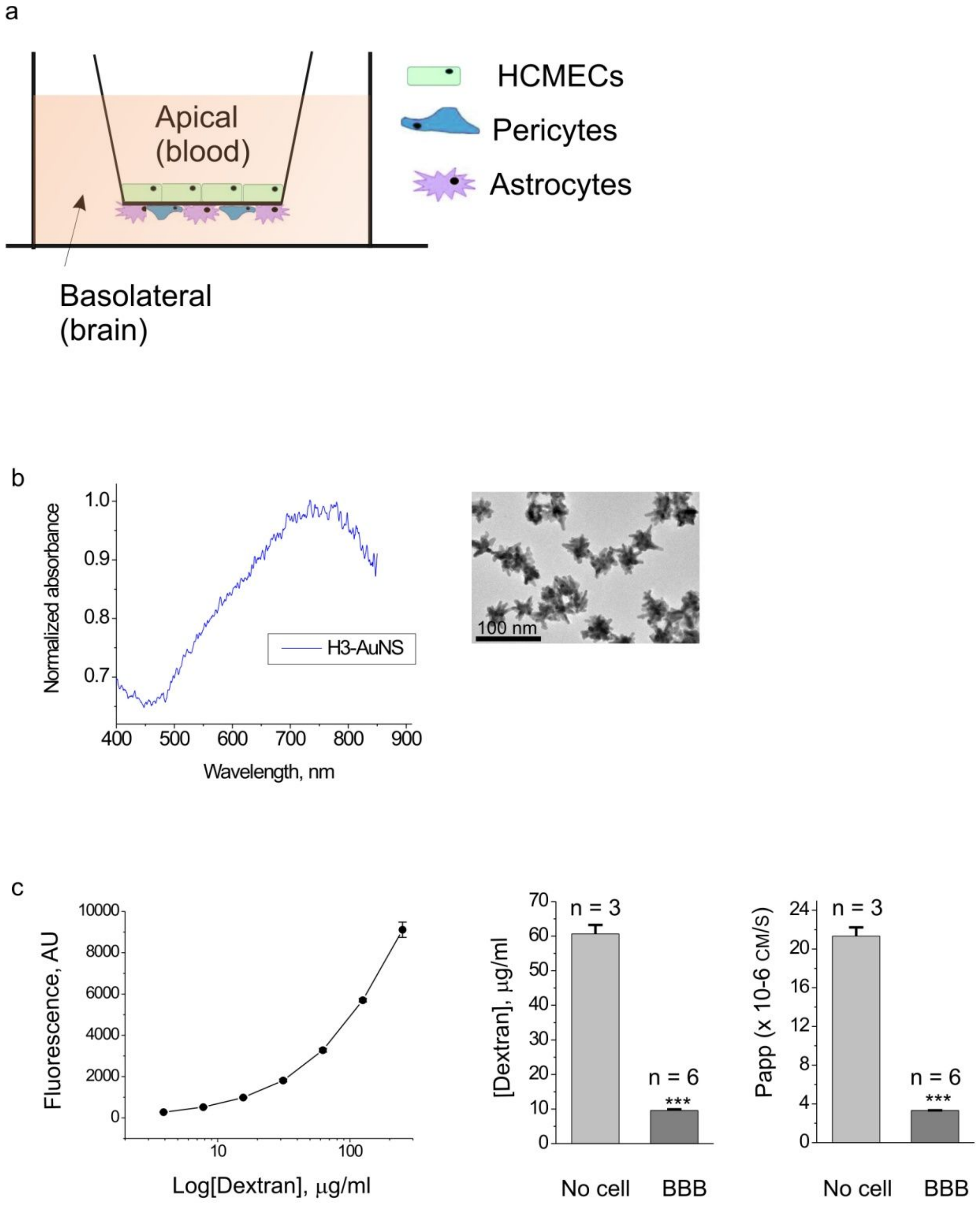

Figure S5: (a**)** Schematic representation of the BBB model. Human brain endothelial cells (HCMECs) are seeded on the apical side of the insert and a mixed culture of astrocytes and pericytes on the basolateral side of the insert. (b) UV-vis absorbance of H3-conjugated nanostars (inset) for BBB studies. (c) Left, calibration curve for dextran-rhodamine. Right, dextran concentrations in the basolateral compartment and permeability coefficients calculated from the diffusion of 70 kDa dextran-rhodamine (500 µg/ml) through a membrane (No cell) and a three-component transwell BBB model. ***$p < 0.001$ *vs*. No cell, *Student*t t-test. Data are presented as mean ± SEM.
